# Synthesis of potent and selective HDAC6 inhibitors led to unexpected opening of a quinazoline ring†

**DOI:** 10.1039/d2ra01753a

**Published:** 2022-04-13

**Authors:** Davide Moi, Andrea Citarella, Davide Bonanni, Luca Pinzi, Daniele Passarella, Alessandra Silvani, Clelia Giannini, Giulio Rastelli

**Affiliations:** Department of Life Sciences, University of Modena and Reggio Emilia Via Campi 103 Modena 41125 Italy giulio.rastelli@unimore.it; Department of Chemistry, University of Milan Via Golgi 19 Milano 20133 Italy

## Abstract

Histone deacetylase (HDAC) inhibitors are highly involved in the regulation of many pharmacological responses, which results in anti-inflammatory and anti-cancer effects. In the present work, chemoinformatic analyses were performed to obtain two potent and selective aminotriazoloquinazoline-based HDAC6 inhibitors. We unexpectedly obtained an aminotriazole from a water-driven ring opening of the triazoloquinazoline scaffold. Both compounds were evaluated as HDAC6 inhibitors, resulting in subnanomolar inhibitory activity and high selectivity with respect to class I HDAC1 and HDAC8. Importantly, the compounds were about 3- and 15-fold more potent compared to the reference compound trichostatin A. Additionally, the predicted binding modes were investigated with docking. Considering that the aminotriazole scaffold has never been embedded into the chemical structure of HDAC6 inhibitors, the present study suggests that both the aminotriazoloquinazoline and aminotriazole classes of compounds could be excellent starting points for further optimization of potential anticancer compounds, introducing such novel groups into a relevant and new area of investigation.

## Abstract 2

Potent and selective inhibitors of HDAC6 have been designed, synthesized and tested. An unexpected opening of the quinazoline ring led to both aminotriazoloquinazoline and aminotriazole compounds with potent activity and isoform selectivity.

## Graphical abstract

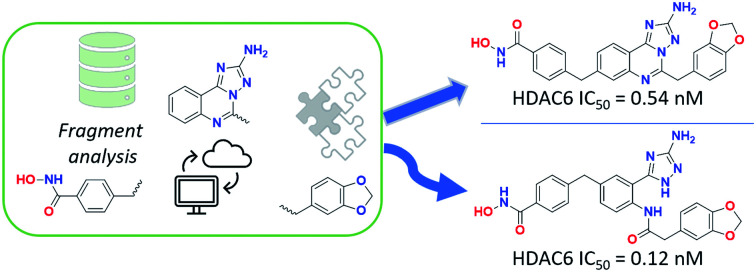

## Introduction

**Fig. 1:**
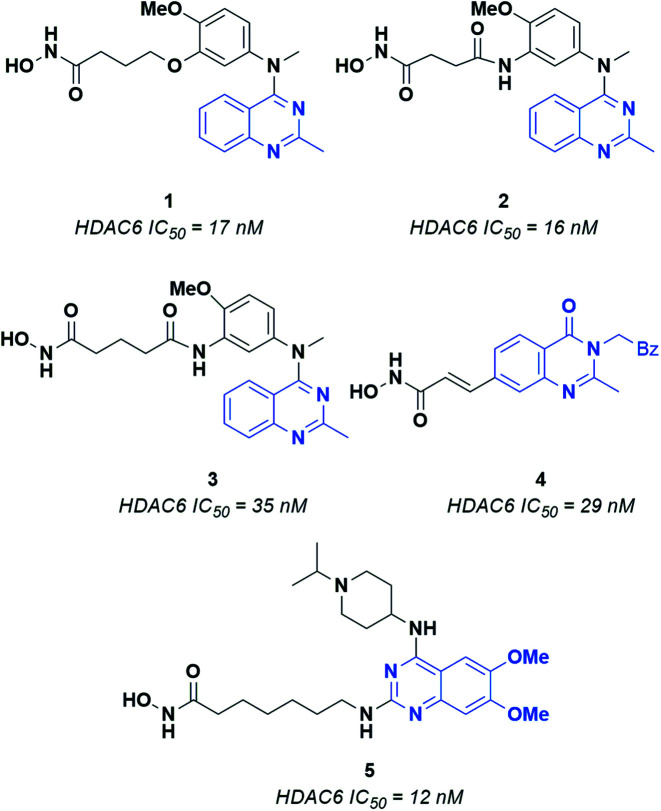
Known quinazoline-capped inhibitors of HDAC6.

Epigenetic regulation is generally defined as the change in gene function deriving from DNA modification operated through chromatin remodeling, RNA regulation and histone modification. Several enzymes are involved in epigenetic regulation.^1^ Histone deacetylases (HDACs) play a crucial role in this process, catalyzing the removal of acetyl groups from lysine residues in histone and several non-histone proteins.^2–4^ Currently, eighteen HDACs have been identified and grouped into four different classes, according to their cellular localization and enzymatic activity. Class I HDACs includes four isoforms (*i.e.*, HDAC1, 2, 3, 8), which are mainly located in the nucleus and can act on histones and transcription factors. Class II HDACs consists of two subclasses, *i.e.*, IIa (HDAC4, 5, 7, 9) and IIb (HDAC 6 and 10). Of note, several members of class II HDACs can shuttle between the nucleus and the cell cytoplasm, where they can modulate the activity of various non-histone proteins. Class III histone deacetylases includes seven isoforms named sirtuins (SIRT1-7), which present several differences with respect to the other HDACs. Moreover, the activity of sirtuins is mainly involved in the regulation of metabolic processes as insulin secretion, ammonia detoxification and metabolic inflammation.^5^ Class IV includes only HDAC11, which is involved in the regulation of many biological processes in cells.^6^ Classes I, II and IV require Zn^2+^ for catalysis, while sirtuins employ NAD^+^ (nicotinamide adenine dinucleotide) as a cofactor.^3^ Recent studies have shown that Zn-dependent HDACs, and especially class I and class IIb isoforms, are overexpressed in many types of tumors, such as breast and liver cancer, multiple myeloma and neuroblastoma.^7^ HDAC6 plays a crucial role in protein degradation, cell shape, migration and regulation of immunomodulatory factors^8^ and is a well-established target for the development of anticancer compounds. Interestingly, the HDAC6 inhibitors reported so far share recurring features: (i) a zinc binding group (ZBG), which forms a stable coordination complex with the catalytic Zn^2+^ ion; (ii) a hydrophobic linker that fits into the catalytic tunnel of the active site, and; (iii) a CAP moiety interacting with the residues that line the entrance of the pocket.^9,10^ This latter region has been extensively probed for the design of HDAC6 selective inhibitors. Indeed, HDAC6 presents a CAP region significantly different in terms of shape and volume with respect to that of the other Zn-dependent histone deacetylases. As for the ZBG, the most relevant and explored one is the hydroxamic acid (HA), which is a powerful chelator of Zn^2+^. The formation of HA–Zn^2+^ complexes generally occur *via* a bidentate mechanism, resulting in significant enzyme inhibition.^10^ Several structurally different CAP groups have been explored for the design of HDAC6 inhibitors, the quinazoline motif demonstrating to be a valuable scaffold to obtain potent inhibitors. A few key examples of HDAC6 inhibitors bearing the quinazoline scaffold are reported in Fig. 1.^11–14^ For example, compounds 1, 2 and 3 consist of a quinazoline core decorated at position 4 with a substituted aniline, which in turn is connected to an aliphatic chain linked to the ZBG through ethereal (1) or amidic (2 and 3) bonds. Changing the position of the ZBG led to 4, a potent cinnamyl derivative. The most significant structural modification was obtained by switching the ZBG to position 2, affording 5, which, to the best of our knowledge, is the most active quinazoline inhibitor reported so far (IC_50_ = 12 nM).^13^

Based on these premises and relying on our previous work on HDAC6 inhibitors,^2,15–19^ in this study we report the computational design and the synthesis of two structurally novel derivatives containing an aminotriazoloquinazoline (11a) and aminotriazole scaffold (18). The two synthesized compounds were tested *in vitro* to evaluate their inhibitory activity against HDAC6. Docking investigations were performed to provide structural explanations of the observed inhibitory activity. Of note, this is the first study that explores the aminotriazoloquinazoline and aminotriazole scaffolds as CAP groups of HDAC6 inhibitors. Moreover, for the first time we observed the opening of the triazoloquinazoline ring in water, which was previously investigated only in the presence of carbon nucleophiles. The results are particularly appealing in light of the potent sub-nanomolar HDAC6 inhibitory activity displayed by the two compounds.

## Results and discussion

### Design of aminotriazoloquinazoline compounds based on chemoinformatics analyses

**Fig. 2:**
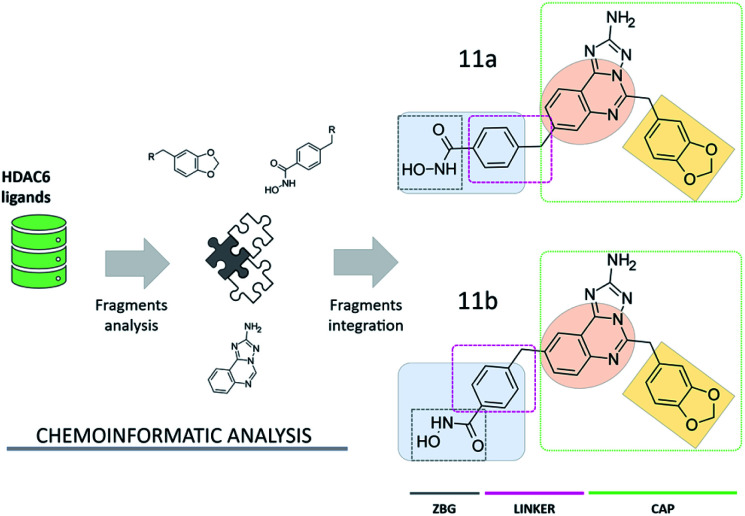
Design of aminotriazoloquinazoline-based HDAC6 inhibitors. The 1,3-benzodioxole (light orange) and aminotriazoloquinazoline (dark orange) moieties represent the CAP group of the ligands.

Compound 11a (Fig. 2) was assembled from a set of chemical scaffolds selected among those more frequently occurring in potent HDAC6 inhibitors, by adopting an approach similar to that of our previous studies.^20^ To this aim, HDAC6 inhibitors collected from ChEMBL were firstly fragmented as detailed in the ESI,† identifying a set of substructures frequently present in the active and inactive compounds. Then, the identified chemical fragments were ranked according to their frequency of occurrence in active over inactive compounds. This allowed us to identify the benzohydroxamate, the 1,3-benzodioxole and the quinazoline moieties as valuable building blocks for the design and assembly of novel HDAC6 inhibitors (see Table S1† and Fig. 2). Interestingly, the benzohydroxamate moiety is frequently present in HDAC6 inhibitors. The hydroxamate coordinates the Zn^2+^,^10^ while the phenyl ring establishes favorable interactions with two phenylalanine residues of HDAC6 flanking the catalytic tunnel. Moreover, the 1,3-benzodioxole moiety has been explored for the optimization of structurally diverse classes of HDAC6 inhibitors, with promising results.^21–23^ The three chemical moieties emerging from these analyses were assembled into compound 11a, a substituted aminotriazoloquinazoline derivative. The connectivity between the three fragments was inspired by information available from HDAC6 crystal structures in complex with ligands and then verified by means of extensive similarity estimations (see ESI†) and docking (see below). Interestingly, this is the first time that the aminotriazoloquinazoline group is explored for the design of HDAC6 inhibitors. Of note, a visual inspection of the identified molecular fragments suggested that the benzohydroxamate moiety could be integrated in either position 8 (11a, Fig. 2) or 9 (11b, Fig. 2) of the aminotriazoloquinazoline group, without potentially altering the HDAC6 inhibitory activity.

### Chemistry

**Scheme 1:**
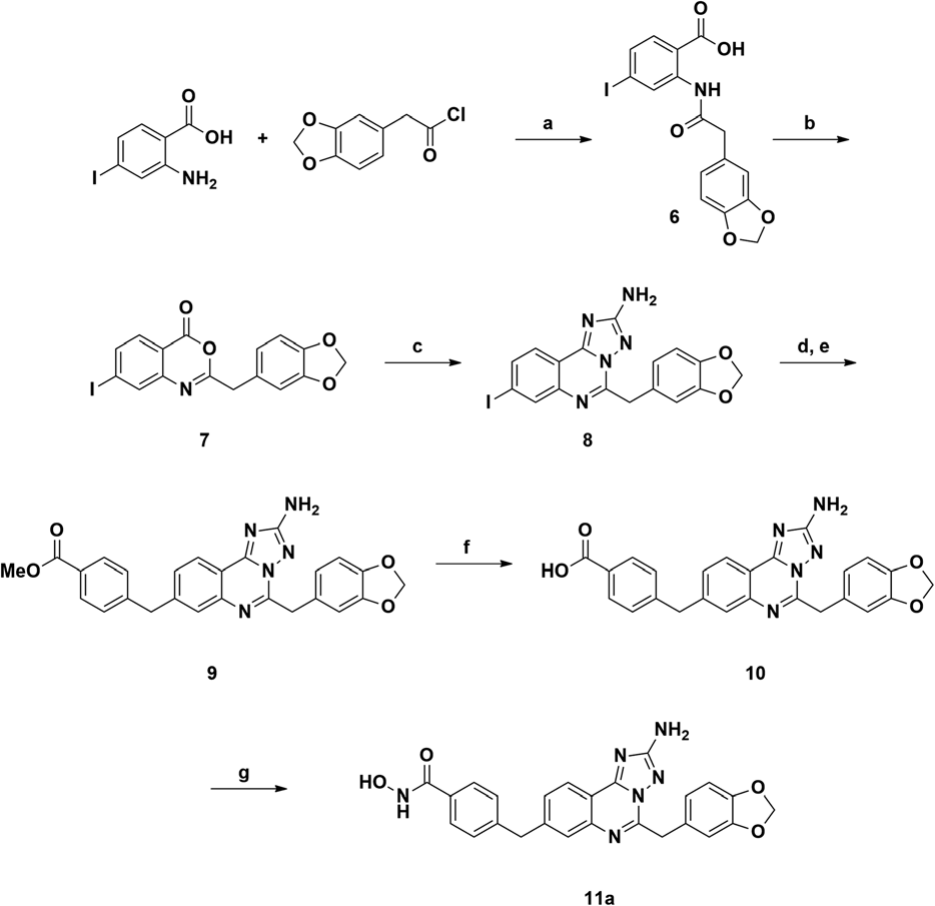
Reagents and conditions: (a) NEt_3_, DCM, rt, overnight, yield 98%; (b) Ac_2_O, 150 °C, 10 min, yield 74%; (c) aminoguanidine bicarbonate, pyridine, 180 °C, microwave, 20 min, yield 93%; (d) bis(pinacolato)diboron, Pd(dppf)Cl_2_, AcOK, DMF, 120 °C, 4 h; (e) methyl 4-(bromomethyl)benzoate, Pd(PPh_3_)_4_, K_2_CO_3_, toluene/EtOH 3 : 1 (v/v), 120 °C, 4 h, yield 70%; (f) LiOH, 1,4-dioxane/water 1 : 1 (v/v), rt, overnight, yield 95%; (g) NH_2_OTMS, HOBt, EDCI, DIPEA, DMF, rt, overnight, 51%.

The synthetic route to compound 11a was designed following a linear approach based on the construction of the aminotriazoloquinazoline core, a subsequent Suzuki–Miyaura cross-coupling for insertion of the benzylic linker and a final functional group interconversion to introduce the hydroxamic acid moiety (Scheme 1).

Starting from the commercially available 4-iodoanthranilic acid and 2-(benzo[*d*][1,3]dioxol-5-yl)acetyl chloride, the amide 6 was achieved in excellent yield, after precipitation with HCl. Treatment of 6 with acetic anhydride at reflux for 10 min gave quantitatively benzoxazinone intermediate 7, which was afterward reacted with aminoguanidine bicarbonate under microwave irradiation to provide the aminotriazoloquinazoline 8 in high yield.^24^ Several conditions for the conversion of the iodo derivative 8 into the corresponding pinacolate boronic ester were evaluated, as reported in Table 1. The use of Pd(PPh_3_)_4_ and the combination of Pd(OAc)_2_-PPh_3_ in dry toluene led to starting material recovery (entries 1–2), as well as the use of Pd(PPh_3_)_4_ in 1,4-dioxane/water 1 : 1 (v/v) or PdCl_2_(PPh_3_)_2_ in dry 1,4-dioxane (entries 3–4). The formation of the desired product was observed using Pd(dppf)Cl_2_ in 1,4-dioxane (Entry 5), while switching the solvent to dry DMF improved the conversion up to 63%, as calculated by ^1^H NMR (Entry 6). The crude mixture was analysed by LC-MS, which highlighted the predominance of the desired pinacolate boronic ester, together with a low percentage of the free boronic acid. The mixture was filtered off, the solvent was evaporated under reduced pressure and the crude was used in the following step without further purification. The Suzuki–Miyaura cross-coupling with methyl 4-bromomethyl benzoate, using Pd(PPh_3_)_4_ and K_2_CO_3_ in toluene/EtOH 3 : 1 (v/v), afforded the desired product 9 in good yield, after column chromatography purification. Methyl ester 9 was hydrolysed under basic conditions by aqueous LiOH and coupled with *O*-trimethylsilylhydroxylamine. To our advantage, coupling and –OH deprotection occurred in a single step operation, providing the final product 11a, after reverse phase column chromatography purification, with good yield.

**Table 1:** Summary of the reaction conditions to obtain 9

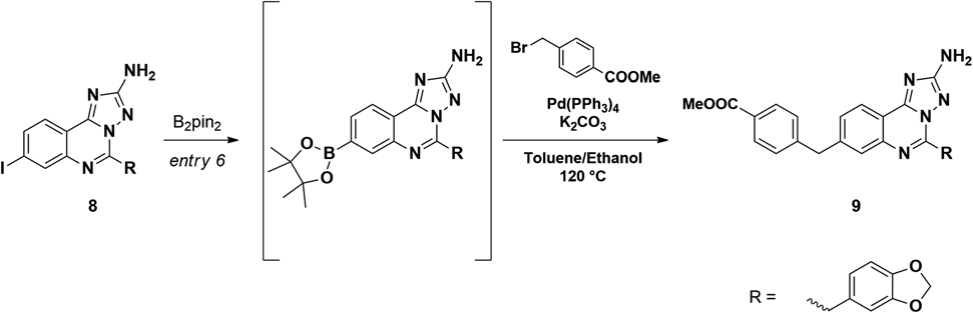					
Entry	Solvent	Cat./ligand	Temp.	Time	Yield (9)
1	Toluene	Pd(OAc)_2_-PPh_3_	110 °C	6 h	—
2	Toluene	Pd(PPh_3_)4	120 °C	6 h	—
3	1,4-Dioxane/water	Pd(PPh_3_)_4_	120 °C	6 h	—
4	1,4-Dioxane	PdCl_2_(PPh_3_)_2_	110 °C	7 h	—
5	1,4-Dioxane	Pd(dppf)Cl_2_	110 °C	6 h	—
**6**	**DMF**	**Pd(dppf)Cl**_**2**_	**120 °C**	**4 h**	**70**

**Scheme 2:**
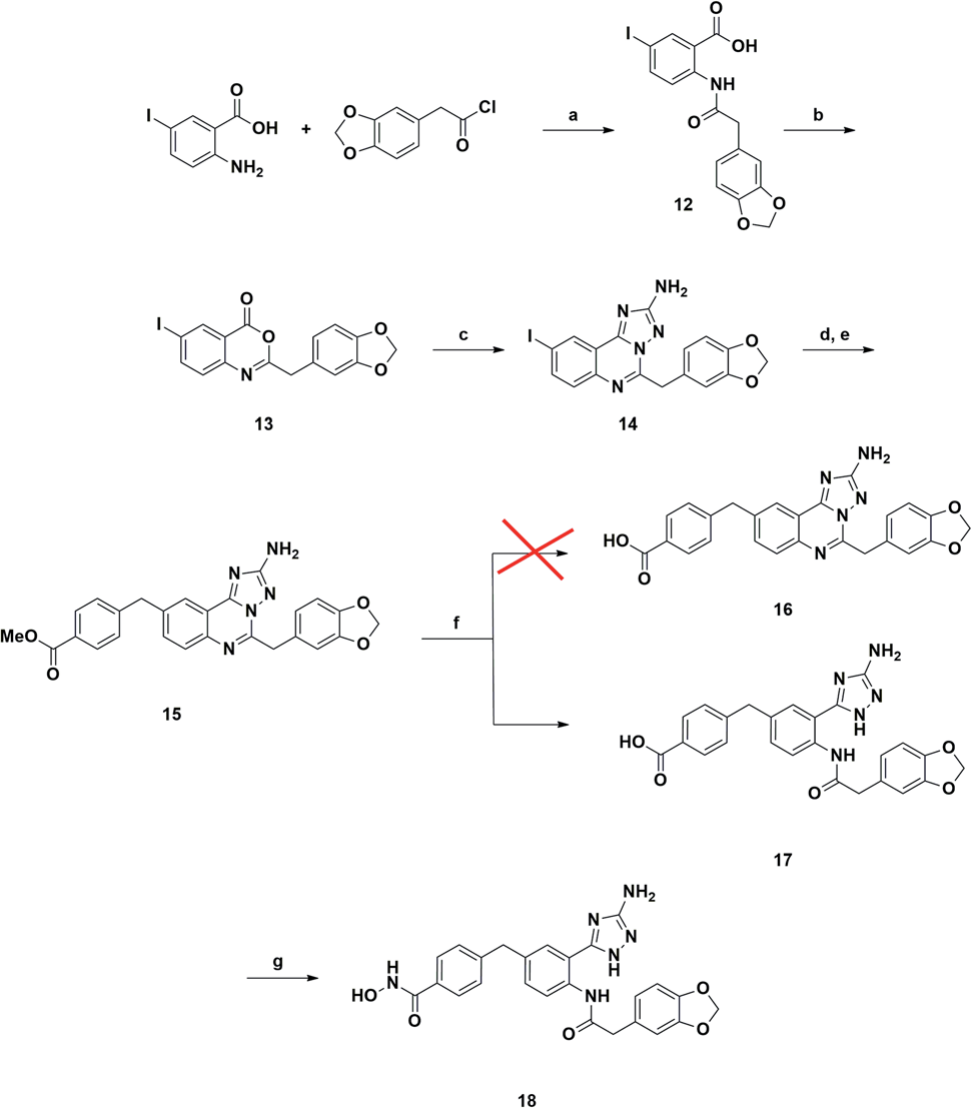
Reagents and conditions: (a) NEt_3_, DCM, rt, overnight, yield 93%; (b) Ac_2_O, 150 °C, 10 min, yield 84%; (c) aminoguanidine bicarbonate, pyridine, 180 °C, microwave, 20 min, yield 93%; (d) Bis(pinacolato)diboron, Pd(dppf)Cl_2_, AcOK, DMF, 120 °C, 4 h; (e) methyl 4-(bromomethyl)benzoate, Pd(PPh_3_)_4_, K_2_CO_3_, toluene/EtOH 3 : 1 (v/v), 120 °C, 4 h, yield 68%; (f) LiOH, 1,4-dioxane/water 1 : 1 (v/v), rt, overnight, yield 86%; (g) NH_2_OTMS, HOBt, EDCI, DIPEA, DMF, rt, overnight, yield 43%.

A synthetic strategy similar to that described above was adopted in the attempt to prepare the 9-substituted derivative (Scheme 2). However, the same hydrolysis conditions (step f) did not lead to the desired product 16. LC-MS analysis revealed the exclusive presence of a molecular structure (17) showing *m*/*z* [M + 18]^+^ with respect to the product of interest, suggesting the addition of water and the concomitant ring opening of the quinazoline ring. Interestingly this result has never been reported in the literature, although several examples of triazoloquinazoline ring opening have been observed only in the presence of carbon nucleophiles.^25–27^ Any attempt to discourage this side reaction, *e.g.*, by carefully monitoring the reaction course and also reducing temperature to 0 °C, proved to be ineffective. Reasoning that the obtained acid derivative 17 could also be interesting for biological evaluation, we proceeded to convert it into the final hydroxamic acid 18, whose chemical structure was confirmed by two-dimensional NMR analysis.

### *In vitro* inhibitory activity

The synthetized compounds 11a and 18 were tested *in vitro* to assess their inhibitory activity on purified recombinant HDAC6 enzyme. The results are reported in Table 2.

**Table 2:** *In vitro* inhibitory activity (IC_50_, nM) of compounds 11a and 18 toward human HDAC6, HDAC1 and HDAC8

Compound	IC_50_ (nM)		
	HDAC6	HDAC1	HDAC8
11a	0.5	6080	653
18	0.1	1820	1560
Trichostatin A	1.9	3.25	648

Both 11a and 18 displayed potent, subnanomolar inhibitory activity towards HDAC6, resulting about 3- and 15-fold more potent than the reference compound trichostatin A (Table 2). Intriguingly, the unplanned compound 18 showed potent HDAC6 inhibitory activity. This result is particularly appealing, considering that aminotriazole compounds have never been reported as HDAC6 inhibitors. Furthermore, 11a and 18 were tested *in vitro* against purified recombinant HDAC1 and HDAC8 to evaluate their selectivity profile. Compound 11a resulted more than 12 000-fold and 1000-fold selective for HDAC6 with respect to HDAC1 and HDAC8, respectively, while 18 was even more selective, being than 18 000-fold and 15 000-fold more active on HDAC6 than HDAC1 and HDAC8, respectively (Table 2). Consequently, our study suggests that both the aminotriazoloquinazoline and aminotriazole classes of compounds are novel and excellent starting points for further optimization for the development of highly potent and selective HDAC6 inhibitors.

### Molecular docking in the HDAC6 active site

**Fig. 3:**
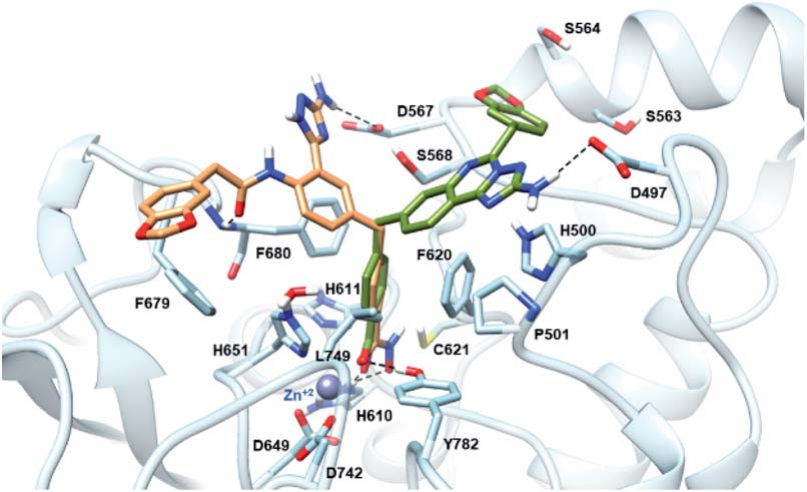
Binding mode predicted for compound 11a (sticks, coloured in green) and 18 (sticks, coloured in sienna) into the HDAC6 binding site (PDB ID 5EDU). The residues lining the enzyme binding site are shown in light blue sticks. The catalytic Zn^2+^ ion is depicted as a grey sphere, while the hydrogen bonds are shown as black dashed lines.

The newly synthesized compounds were docked into a representative crystal structure of HDAC6 as detailed in the ESI,† to evaluate whether they provide favorable docking scores and a binding mode consistent with those reported in crystal structure complexes. The complementarity of 11a and 18 with the HDAC6 binding site was thus evaluated through docking calculations performed on the 5EDU crystal structure of the human HDAC6 enzyme.^28^ For both ligands, we found that the ionized hydroxamate group coordinates the Zn^2+^ in bidentate mode,^28^ and it accepts two hydrogen bonds from Y782 and H610 through the carbonyl and the deprotonated hydroxyl, respectively (Fig. 3). Moreover, the phenyl ring of 11a and 18 establishes favourable π–π stacking interactions with the side chains of F620 and F680. Interestingly, the CAP groups of 11a and 18 are oriented differently (Fig. 3). The aminotriazoloquinazoline moiety of compound 11a stretches over F620 to donate a hydrogen bond to the side chain of D497, and the 1,3-benzodioxole accommodates in proximity of S564 and S568. On the contrary, the CAP group of 18 is directed towards F680, the amide carbonyl hydrogen bonds with the backbone nitrogen of F680, the 1,3-benzodioxole moiety forms a T-shaped stacking with the phenyl ring of F679, and the NH_2_ of the triazole hydrogen bonds with the side chain of D567. Compounds 11a and 18 achieved favourable docking scores of −10.4 and −11.0 kcal mol^−1^, respectively, compared to the self-docking result of −8.6 kcal mol^−1^.

## Conclusions

In conclusion, a series of chemoinformatic analyses performed on reported histone deacetylase 6 inhibitors allowed the identification of the benzohydroxamate, 1,3-benzodioxole and quinazoline scaffolds as key moieties which were combined for the design of novel aminotriazoloquinazoline-based HDAC6 inhibitors. Two compounds were synthesized and tested *in vitro* to assess their inhibitory activity on purified recombinant HDAC6 enzyme. Aminotriazoloquinazoline 11a inhibited the enzyme (IC_50_ = 0.5 nM) 3 times more potently than the reference compound, while the unexpected ring-opened derivative 18 was even more active (IC_50_ = 0.1 nM), being about 15 times more active compared to thricostatin A. In addition, both 11a and 18 displayed high selectivity towards HDAC6 compared with the class I isoforms HDAC1 and HDAC8. The results indicate that the aminotriazoloquinazoline and aminotriazole scaffolds stand out as new starting points for the development of HDAC6 inhibitors, ranking in a promising area for future investigations.

## Experimental

### Chemoinformatic analyses

Histone deacetylase 6 (HDAC6) inhibitors with activity data reported as IC_50_, *K*_i_, *K*_d_, EC_50_ were firstly downloaded from the ChEMBL database (https://www.ebi.ac.uk/chembl/, accessed on: September 28^th^, 2021).^29^ Then, duplicate records deriving from multiple assays on the same target were removed, retaining those with the best activity value. This allowed to obtain 3582 unique ligands, 2304 and 1278 compounds of which have reported activity data below 1 μM (herein labeled as “actives”) and higher than 1 μM (classified as “inactives”), respectively. Afterwards, an analysis of the molecular fragments composition was performed for the collected HDAC6 inhibitors. To this aim, the ligands were fragmented according to the BRICS,^30^ Bemis–Murcko^31^ and Recap^32^ algorithms implemented in the RDKit^33^ and OpenEye python toolkits,^34^ and by using Chomp (version 3.1.1.2, OpenEye)^35^ with default settings. Fragments whose substructure was not included in at least three active molecules and duplicates derived by the different fragmentation algorithms were discarded. Moreover, chemical moieties with a number of atoms lower than 4 or higher than 12 were also removed, obtaining a total of 544 unique fragments. The generated fragments were then used as queries for the identification of the substructures that are more frequently present in already reported HDAC6 compounds. The most interesting substructures emerging from the analysis and their related fragments (Table S2, in ESI†) were used as building blocks for the design of novel candidate compounds. The similarity degree between compound 11a, 11b and HDAC6 inhibitors extracted from ChEMBL was then evaluated by means of 2D fingerprints-based estimations. Similarity estimations were performed with MACCS and ECFP4 fingerprints (fp) implemented in RDKit python toolkits (https://www.rdkit.org),^33^ with settings consistent to those employed in our previous studies.^36,37^ Similarity records with Tanimoto coefficients below 0.8 and 0.3 for MACCSfp and ECFP4fp, respectively, were discarded. A visual inspection of the best-ranking ligand-based records (Table S1, in ESI†) was finally performed to evaluate whether compound 11a and 11b present key structural features and connectivity characterizing potent HDAC6 inhibitors, while showing a reasonable degree of chemical novelty.

### Docking

All the analyses were conducted on the 5EDU crystal structure of the human HDAC6 protein.^28^ The crystal structure of the protein underwent an optimization process using the Protein Preparation Wizard tool, implemented in Maestro of the Schrödinger Suite (release 2021-1).^38^ Missing hydrogen atoms were added, and bond orders were assigned. The prediction of protonation states for the protein residues was accomplished by using PROPKA, with the pH set to 7.4. Docking studies were performed by using XP protocol of the Glide program,^39^ keeping the ligands flexible. Default settings were used for the analyses and metal coordination constraint was applied during docking procedure to allow the ligands to coordinate to the catalytic Zn^2+^ ion.

### *In vitro* assay

*In vitro* tests on HDAC6, HDAC1 and HDAC8 were performed at Reaction Biology Corp. with modalities described below. The enzyme reactions were carried out in a solution of 50 mM Tris–HCl (pH 8.0), 137 mM NaCl, 2.7 mM KCl, 1 mM MgCl_2_, and 1 mg ml^−1^ BSA with the fluorogenic peptide from p53 residues 379–382 (RHKK-Ac-AMC) substrate for HDAC6 and HDAC1, and the fluorogenic peptide from p53 residues 379–382 (RHK-Ac-K-Ac-AMC) for HDAC8. The compounds were dissolved in DMSO and tested with 3-fold serial dilution starting from 100 μM. The enzymatic assays were performed by adding HDAC6 and the ligand solutions in the reaction buffer described above. After reaction termination, the fluorescence signal (Ex 360 nm/Em 460 nm) was recorded (in around 30 minutes) by adding a volume of 2 μM trichostatin A, 16 mg mL^−1^ trypsin in 50 mM Tris–HCl, pH 8.0, 137 mM NaCl, 2.7 mM KCl, 1 mM MgCl_2_. IC_50_ values were calculated by using the GraphPad Prism 4 program based on a sigmoidal dose–response equation. The blank (DMSO) value concentration was entered as 1.00 × 10^−12^ M for curve fitting. Trichostatin A was used as a reference compound.

### General experimental procedures

Unless otherwise stated, reagents and solvents were purchased from Merck (Milan, Italy), Fluorochem (Hadfield, United Kingdom) or TCI (Zwijndrecht, Belgium) and used without further purification. All reactions were carried out in oven-dried glassware and dry solvents, under nitrogen atmosphere and were monitored by TLC on silica gel (Merck precoated 60 F_254_ plates), with detection by UV light (254 nm) or by permanganate, or by HPLC. HPLC was performed on Agilent 1100 Series System. Products were purified by flash column chromatography, using silica gel Merck 60 (230–400 mesh) as stationary phase. ^1^H NMR and ^13^C NMR spectra were recorded on a Bruker Avance Spectrometer (400 MHz), using commercially available deuterated (chloroform-*d*, DMSO-*d*_6_) solvent at room temperature. Chemical shifts are reported in parts per million (*δ* ppm), compared to TMS as an internal standard. Multiplicities in ^1^H NMR are reported as follow: s – singlet, d – doublet, t – triplet, m – multiplet, br – broad. Data for ^13^C NMR are reported in chemical shift (*δ* ppm). High resolution mass spectra (HRMS) were recorded using the Q-ToF Synapt G2-Si HDMS Acquity UPLC I-Class Photodiode Detector Array (PDA) (Waters).

#### 2-(2-(Benzo[*d*][1,3]dioxol-5-yl)acetamido)-4-iodobenzoic acid (6)

To a solution of 2-(benzo[*d*][1,3]dioxol-5-yl)acetyl chloride (967 mg, 4.88 mmol, 1.2 equiv.) in anhydrous dichloromethane (10 mL) was added 2-amino-4-iodobenzoic acid (1.0 g, 4.06 mmol, 1 equiv.). After the addition of triethylamine (2 mL, 16.24 mmol, 4 equiv.), the mixture was left stirring at room temperature overnight. The solvent was removed under reduced pressure and the residue was diluted with 10 mL of 2 N HCl solution. The formed precipitate was filtered, washed with water and dried under vacuum, to afford 6 as a yellow non-crystalline solid (1.7 g, 98% yield). ^1^H NMR (400 MHz, DMSO-*d*_6_): 11.06 (s, 1*H*, OH), 8.97 (s, 1*H*, NH), 7.66 (d, ^3^*J*_H,H_ = 8.5 Hz, 1*H*, Ar H), 7.50 (dd, ^3^*J*_H,H_ = 8.5 Hz, ^4^*J*_H,H_ = 1.5 Hz, 1*H*, Ar H), 6.91 (d, ^4^*J*_H,H_ = 1.5 Hz, 1*H*, Ar H), 6.89–6.80 (m, 3*H*, Ar H), 5.99 (s, 2*H*, O-CH_2_), 3.68 (s, 2*H*, Ar-CH_2_). ^13^C NMR (100 MHz, DMSO-*d*_6_): 170.1, 169.0, 147.4, 146.3, 141.4, 132.4, 131.4, 128.0, 127.9, 122.9, 115.7, 109.9, 108.4, 102.0, 100.9, 44.1. MS (ESI), *m*/*z* [M + H]^+^: 425.87. HRMS (ESI), *m*/*z* [M + H]^+^: calculated for C_16_H_13_INO_5_^+^ 425.9833; found 425.9837. HPLC rt: 19.00 min.

#### 2-(Benzo[*d*][1,3]dioxol-5-ylmethyl)-7-iodo-4*H*-benzo[*d*][1,3]oxazin-4-one (7)

A stirred solution of 6 (1.7 g, 4.0 mmol, 1 equiv.) in acetic anhydride (15 mL) was refluxed for 10 min. The solvent was removed under reduced pressure and the residue was taken up in diethyl ether. The formed precipitate was filtered and dried under vacuum to afford 7 as a white non-crystalline solid (1.2 g, 74% yield). ^1^H NMR (400 MHz, DMSO-*d*_6_): 7.99 (d, ^4^*J*_H,H_ = 1.5 Hz, 1*H*, Ar H), 7.94 (dd, ^3^*J*_H,H_ = 8.5 Hz, ^4^*J*_H,H_ = 1.5 Hz, Ar H), 7.87 (d, ^3^*J*_H,H_ = 8.5 Hz, 1*H*, Ar H), 6.94 (d, ^4^*J*_H,H_ = 1.4 Hz, 1*H*, Ar H), 6.88 (d, 1*H*, ^3^*J*_H,H_ = 7.7 Hz, Ar H), 6.84 (dd, ^3^*J*_H,H_ = 7.7 Hz, ^4^*J*_H,H_ = 1.4 Hz Ar H), 6.00 (s, 2*H*, O-CH_2_), 3.93 (s, 2*H*, Ar-CH_2_-Ar). ^13^C NMR (100 MHz, DMSO-*d*_6_): 162.3, 159.0, 147.4, 146.6, 146.4, 137.3, 134.8, 129.2, 128.0, 122.6, 116.1, 109.7, 108.3, 105.4, 101.0, 40.0. MS (ESI), *m*/*z* [M + H]^+^: 408.12. HRMS (ESI), *m*/*z* [M + H]^+^: calculated for C_16_H_11_INO_4_^+^ 407.9728; found 407.9728. HPLC rt: 21.0 min.

#### 5-(Benzo[*d*][1,3]dioxol-5-ylmethyl)-8-iodo-[1,2,4]triazolo[1,5-*c*]quinazolin-2-amine (8)

A solution of 7 (1.0 g, 2.4 mmol, 1 equiv.) and aminoguanidine bicarbonate (359 mg, 2.6 mmol, 1.1 equiv.) in anhydrous pyridine (4 mL) was heated under microwave condition at 180 °C for 30 min. The reaction mixture was cooled to room temperature and diluted with water/methanol 3 : 1 (v/v). The formed precipitate was filtered, washed with a solution of water/methanol 3 : 1 (v/v) and dried under vacuum to afford the desired 8 as a white non-crystalline solid (1.0 g, 93% yield). ^1^H NMR (400 MHz, DMSO-*d*_6_): 8.26 (s, 1*H*, Ar H), 7.95 (s, 2*H*, Ar H), 6.98 (s, 1*H*, Ar H), 6.83 (s, 2*H*, Ar H), 6.56 (s, 2*H*, NH_2_), 5.97 (s, 2*H*, O-CH_2_), 4.41 (s, 2*H*, Ar-CH_2_-Ar). ^13^C NMR (100 MHz, DMSO-*d*_6_): 165.8, 159.1, 148.7, 147.2, 146.9, 143.2, 136.0, 130.0, 129.0, 127.8, 122.4, 114.7, 109.7, 108.2, 100.9, 98.3, 37.9. MS (ESI), *m*/*z* [M + H]^+^: 446.16. HRMS (ESI), *m*/*z* [M + H]^+^: calculated for C_17_H_13_IN_5_O_2_^+^ 446.0109; found 446.0110. HPLC rt: 17.9 min.

#### Methyl 3-((2-amino-5-(benzo[*d*][1,3]dioxol-5-ylmethyl)-[1,2,4]triazolo[1,5-*c*]quinazolin-8-yl)methyl)benzoate (9)

An oven-dried flask was charged with 8 (1.0 g, 2.2 mmol, 1 equiv.), bis(pinacolato)diboron (614 mg, 2.4 mmol, 1.1 equiv.), Pd(dppf)Cl_2_ (14 mg, 0.13 mmol, 0.06 equiv.), AcOK (259 mg, 2.6 mmol, 1.2 equiv.) and dry DMF (10 mL). The reaction mixture was purged with N_2_ and then heated to 120 °C. Progress of the reaction was monitored by TLC. After disappearance of the starting material, the reaction mixture was cooled down, filtered off, and the filtrate was concentrated under vacuum. Therefore, the resulting crude was added to a mixture of methyl 4-bromomethyl benzoate (554 mg, 2.4 mmol, 1.1 equiv.), Pd(PPh_3_)_4_ (508 mg, 0.44 mmol, 0.2 equiv.), K_2_CO_3_ (760 mg, 5.5 mmol, 2.5 equiv.) in toluene/EtOH (3 : 1 v/v, 20 mL) and the reaction was heated to 120 °C. After 4 h, the suspension was filtered, and the filtrate was concentrated under reduced pressure. The crude was taken up in ethyl acetate (20 mL) and washed with brine (3 × 15 mL). The organic layer was dried with anhydrous Na_2_SO_4_, filtered and evaporated to give the crude, which was purified by column chromatography using *n*-hexane/ethyl acetate (1 : 1, v/v) as eluent to afford 9 as a white solid (719 mg, 70%); mp. = 227–232 °C. ^1^H NMR (400 MHz, DMSO-*d*_6_): 8.12 (d, ^3^*J*_H,H_ = 8.2 Hz, 1*H*, Ar H), 7.90 (d, ^3^*J*_H,H_ = 8.0 Hz, 2*H*, Ar H), 7.74 (s, 1*H*, Ar H), 7.54 (d, ^3^*J*_H,H_ = 8.2 Hz, 1*H*, Ar H), 7.45 (d, ^3^*J*_H,H_ = 8.0 Hz, 2*H*, Ar H), 6.98 (s, 1*H*, Ar H), 6.83 (s, 2*H*, Ar H), 6.46 (s, 2*H*, NH_2_), 5.96 (s, 2*H*, O-CH_2_), 4.41 (s, 2*H*, Ar-CH_2_-Ar), 4.23 (s, 2*H*, Ar-CH_2_-Ar), 3.82 (s, 3*H*, OCH_3_). ^13^C NMR (100 MHz, DMSO-*d*_6_): 163.7, 150.4, 148.0, 147.9, 146.0, 143.6, 142.0, 140.1, 133.8, 131.2, 129.1, 129.0, 128.7, 128.6, 123.0, 122.7, 115.3, 110.0, 107.4, 101.1, 52.2, 42.0, 38.1. MS (ESI), *m*/*z* [M + H]^+^: 468.29. HRMS (ESI), *m*/*z* [M + H]^+^: calculated for C_26_H_22_N_5_O_4_^+^ 468.1667; found 468.1664. HPLC rt: 19.6.

#### 4-((2-Amino-5-(benzo[*d*][1,3]dioxol-5-ylmethyl)-[1,2,4]triazolo[1,5-*c*]quinazolin-8-yl)methyl)benzoic acid (10)

To a solution of 9 (700 mg, 1.5 mmol, 1 equiv.) in 1,4-dioxane/water (3 : 1, v/v, 12 mL) was added LiOH (287 mg, 12 mmol, 8 equiv.) and the mixture was left stirring at room temperature overnight. The reaction was concentrated under reduced pressure, the residue was taken up in water and adjusted to pH 4 with 2 N HCl. The formed precipitate was filtered, washed with water and dried under vacuum to afford 10 as a white non-crystalline solid (650 mg, 95%). ^1^H NMR (400 MHz, DMSO-*d*_6_): 8.12 (d, ^3^*J*_H,H_ = 8.2 Hz, 1*H*, Ar H), 7.89 (m, ^3^*J*_H,H_ = 8.0 Hz, 2*H*, Ar H), 7.75 (s, 1*H*, Ar H), 7.55 (dd, ^3^*J*_H,H_ = 8.6 Hz, ^4^*J*_H,H_ = 1.5 Hz, 1*H*, Ar H), 7.45 (m, ^3^*J*_H,H_ = 8.0 Hz, 2*H*, Ar H), 6.98 (s, 1*H*, Ar H), 6.83 (s, 2*H*, Ar H), 6.47 (s, 2*H*, NH_2_), 5.97 (s, 2*H*, O-CH_2_), 4.39 (s, 2*H*, Ar-CH_2_-Ar), 4.23 (s, 2*H*, Ar-CH_2_-Ar). ^13^C NMR (100 MHz, DMSO-*d*_6_): 169.0, 157.5, 156.3, 147.2, 146.1, 144.6, 135.1, 134.9, 130.6, 129.3, 129.1, 129.0, 128.6, 127.2, 127.1, 122.4, 119.4, 109.6, 108.1, 100.8, 44.5, 40.1. MS (ESI), *m*/*z* [M + H]^+^: 454.19. HRMS (ESI), *m*/*z* [M + H]^+^: calculated for C_25_H_20_N_5_O_4_^+^ 454.1510; found 454.1506. HPLC rt: 13.79 min.

#### 4-((2-Amino-5-(benzo[*d*][1,3]dioxol-5-ylmethyl)-[1,2,4]triazolo[1,5-*c*]quinazolin-8-yl)methyl)-*N*-hydroxybenzamide (11a)

10 (500 mg, 1.1 mmol, 1 equiv.) and HOBt (178 mg, 1.3 mmol, 1.2 equiv.) were dissolved in dry DMF (8 mL) and DIPEA (0.5 mL, 2.7 mmol, 2.5 equiv.) was added. After 30 min, EDCI (249 mg, 1.3 mmol, 1.2 equiv.) and NH_2_OTMS (0.2 mL, 1.5 mmol, 1.4 equiv.) were added and the mixture was left stirring at room temperature overnight. The reaction mixture was then diluted with ethyl acetate (12 mL) and was washed with saturated aq. solution of NH_4_Cl (3 × 10 mL), saturated aq. solution of NaHCO_3_ (3 × 10 mL) and brine (3 × 10 mL). The organic layer was dried with anhydrous Na_2_SO_4_, filtered and concentrated under reduced pressure. The crude was purified *via* automatic flash column chromatography (reverse phase, water/acetonitrile gradient from 5% to 100%) to afford 11a as a white solid (51% yield, 262 mg); mp. = <267 °C with decomposition. ^1^H NMR (400 MHz, DMSO-*d*_6_): 11.12 (bs, 1*H*, OH), 8.97 (bs, 1*H*, NH), 8.41 (d, ^4^*J*_H,H_ = 1.5 Hz, 1*H*, Ar H), 7.92 (d, ^3^*J*_H,H_ = 8.2 Hz, 1*H*, Ar H), 7.66 (d, ^3^*J*_H,H_ = 8.4 Hz, 2*H*, Ar H), 7.28 (d, ^3^*J*_H,H_ = 8.4 Hz, 2*H*, Ar H), 6.95 (dd, ^3^*J*_H,H_ = 8.2 Hz, ^4^*J*_H,H_ = 1.5 Hz, 1*H*, Ar H), 6.93 (m, 1*H*, Ar H), 6.85 (m, 2*H*, Ar H), 6.34 (bs, 2*H*, NH_2_), 5.97 (s, 2*H*, O-CH_2_), 3.95 (s, 2*H*, Ar-CH_2_-Ar), 3.60 (s, 2*H*, Ar-CH_2_-Ar). ^13^C NMR (100 MHz, DMSO-*d*_6_): 167.2, 165.6, 150.1, 147.5, 147.2, 146.1, 147.8, 141.0, 140.2, 132.7, 129.7, 129.3, 129.2, 127.9, 122.1, 115.4, 109.6, 108.2, 100.9, 40.5, 37.8. MS (ESI), *m*/*z* [M + H]^+^: 469.45. HRMS (ESI), *m*/*z* [M + Na]^+^: calculated for C_25_H_20_N_6_NaO_4_^+^ 491.1438; found 491.1440. HRMS (ESI), *m*/*z* [M + H]^+^: calculated for C_25_H_21_N_6_O_4_^+^ 469.1619; found 469.1620. HPLC rt: 13.04 min.

#### 2-(2-(Benzo[*d*][1,3]dioxol-5-yl)acetamido)-5-iodobenzoic acid (12)

To a solution of 2-(benzo[*d*][1,3]dioxol-5-yl)acetyl chloride (967 mg, 4.88 mmol, 1.2 equiv.) in anhydrous dichloromethane (10 mL) was added 2-amino-5-iodobenzoic acid (1.0 g, 4.06 mmol, 1 equiv.). After the addition of triethylamine (2 mL, 16.24 mmol, 4 equiv.), the mixture was left stirring at room temperature overnight. The solvent was removed under reduced pressure and the residue was diluted with 10 mL of 2 N HCl solution. The formed precipitate was filtered, washed with water and dried under vacuum, to afford 12 as a white non-crystalline solid (1.6 g, 93% yield). ^1^H NMR (400 MHz, DMSO-*d*_6_): 10.98 (s, 1*H*, OH), 8.32 (d, ^3^*J*_H,H_ = 8.9 Hz, 1*H*, Ar H), 8.17 (d, ^4^*J*_H,H_ = 2.2 Hz, 1*H*, Ar H), 7.87 (dd, ^3^*J*_H,H_ = 8.5 Hz, ^4^*J*_H,H_ = 2.2 Hz, 1*H*, Ar H), 6.91 (d, ^4^*J*_H,H_ = 1.2 Hz, 1*H*, Ar H), 6.87 (d, ^3^*J*_H,H_ = 7.9 Hz, 1*H*, Ar H), 6.80 (dd, ^3^*J*_H,H_ = 7.9 Hz, ^4^*J*_H,H_ = 1.2 Hz, 1*H*, Ar H), 5.99 (s, 2*H*, O-CH_2_), 3.66 (s, 2*H*, Ar-CH_2_). ^13^C NMR (100 MHz, DMSO-*d*_6_): 169.9, 168.0, 147.4, 146.3, 142.2, 140.3, 139.0, 128.0, 122.9, 122.0, 118.6, 109.9, 108.4, 100.9, 85.9, 44.1. MS (ESI), *m*/*z* [M + H]^+^: 425.87. HRMS (ESI), *m*/*z* [M + H]^+^: calculated for C_16_H_13_INO_5_^+^ 425.9833; found 425.9836. HPLC rt: 18.78 min.

#### 2-(Benzo[*d*][1,3]dioxol-5-ylmethyl)-6-iodo-4*H*-benzo[*d*][1,3]oxazin-4-one (13)

A stirred solution of 12 (1.6 g, 3.8 mmol, 1 equiv.) in acetic anhydride (15 mL) was refluxed for 10 min. The solvent was removed under reduced pressure and the residue was taken up in diethyl ether. The formed precipitate was filtered and dried under vacuum to afford 13 as a white solid (1.3 g, 84% yield); mp. = 188–190 °C. ^1^H NMR (400 MHz, DMSO-*d*_6_): 8.33 (d, ^4^*J*_H,H_ = 1.8 Hz, 1*H*, Ar H), 8.20 (dd, ^3^*J*_H,H_ = 8.4 Hz, ^4^*J*_H,H_ = 1.8 Hz, Ar H), 7.38 (d, ^3^*J*_H,H_ = 8.4 Hz, 1*H*, Ar H), 6.94 (d, ^4^*J*_H,H_ = 1.3 Hz, 1*H*, Ar H), 6.88 (d, 1*H*, ^3^*J*_H,H_ = 7.5 Hz, Ar H), 6.83 (dd, ^3^*J*_H,H_ = 7.5 Hz, ^4^*J*_H,H_ = 1.3 Hz. Ar H), 6.00 (s, 2*H*, O-CH_2_), 3.93 (s, 2*H*, Ar-CH_2_-Ar). ^13^C NMR (100 MHz, DMSO-*d*_6_): 161.5, 152.8, 147.4, 145.2, 144.8, 136.6, 135.6, 128.3, 127.7, 122.3, 118.3, 109.4, 108.0, 100.7, 92.8, 44.0. MS (ESI), *m*/*z* [M + H]^+^: 408.12. HRMS (ESI), *m*/*z* [M + H]^+^: calculated for C_16_H_11_INO_4_^+^ 407.9728; found 407.9726. HPLC rt: 21.8 min.

#### 5-(Benzo[*d*][1,3]dioxol-5-ylmethyl)-9-iodo-[1,2,4]triazolo[1,5-*c*]quinazolin-2-amine (14)

A solution of 13 (1.0 g, 2.4 mmol, 1 equiv.) and aminoguanidine bicarbonate (359 mg, 2.6 mmol, 1.1 equiv.) in anhydrous pyridine (4 mL) was heated under microwave condition at 180 °C for 30 min. The reaction mixture was cooled to room temperature and diluted with water/methanol 3 : 1 (v/v). The formed precipitate was filtered, washed with a solution of water/methanol 3 : 1 (v/v) and dried under vacuum to afford the desired 14 as a white non-crystalline solid (1.0 g, 93% yield); mp. = 218–222 °C. ^1^H NMR (400 MHz, DMSO-*d*_6_): 8.46 (s, 1*H*, Ar H), 8.05 (d, ^3^*J*_H,H_ = 9.0 Hz, 1*H*, Ar H), 7.65 (d, ^3^*J*_H,H_ = 9.0 Hz, 1*H*, Ar H), 6.98 (s, 1*H*, Ar H), 6.83 (s, 2*H*, Ar H), 6.54 (s, 2*H*, NH_2_), 5.96 (s, 2*H*, O-CH_2_), 4.40 (s, 2*H*, Ar-CH_2_-Ar). ^13^C NMR (100 MHz, DMSO-*d*_6_): 166.8, 148.9, 148.4, 147.2, 145.7, 141.5, 139.8, 131.0, 129.8, 129.0, 122.2, 117.3, 109.7, 108.2, 100.8, 92.7, 37.9. MS (ESI), *m*/*z* [M + H]^+^: 446.15. HRMS (ESI), *m*/*z* [M + H]^+^: calculated for C_17_H_13_IN_5_O_2_^+^ 446.0109; found 446.0104. HPLC rt: 18.6 min.

#### Methyl 3-((2-amino-5-(benzo[*d*][1,3]dioxol-5-ylmethyl)-[1,2,4]triazolo[1,5-*c*]quinazolin-9-yl)methyl)benzoate (15)

An oven-dried flask was charged with 14 (1.0 g, 2.2 mmol, 1 equiv.), Bis(pinacolato)diboron (614 mg, 2.4 mmol, 1.1 equiv.), Pd(dppf)Cl_2_ (14 mg, 0.13 mmol, 0.06 equiv.), AcOK (259 mg, 2.6 mmol, 1.2 equiv.) and dry DMF (10 mL). The reaction mixture was purged with N_2_ and then heated to 120 °C. Progress of the reaction was monitored by TLC. After disappearance of the starting material, the reaction mixture was cooled down, filtered off, and the filtrate was concentrated under vacuum. Therefore, the resulting crude was added to a mixture of methyl 4-bromomethyl benzoate (554 mg, 2.4 mmol, 1.1 equiv.), Pd(PPh_3_)_4_ (508 mg, 0.44 mmol, 0.2 equiv.), K_2_CO_3_ (760 mg, 5.5 mmol, 2.5 equiv.) in toluene/EtOH (3 : 1 v/v, 20 mL) and the reaction was heated to 120 °C. After 4 h, the suspension was filtered, and the filtrate was concentrated under reduced pressure. The crude was taken up in ethyl acetate (20 mL) and washed with brine (3 × 15 mL). The organic layer was dried with anhydrous Na_2_SO_4,_ filtered and evaporated to give the crude, which was purified by column chromatography using *n*-hexane/ethyl acetate (1 : 1, v/v) as eluent to afford 15 as a white solid (702 mg, 68%); mp. = 200–203 °C. ^1^H NMR (400 MHz, CDCl_3_): 8.12 (m, 1*H*, Ar H), 7.96 (d, ^3^*J*_H,H_ = 8.6 Hz, 2*H*, Ar H), 7.88 (s, 1*H*, Ar H), 7.56 (dd, ^3^*J*_H,H_ = 8.7 Hz, ^4^*J*_H,H_ = 2.3 Hz, 1*H*, Ar H), 7.28 (d, ^3^*J*_H,H_ = 8.6 Hz, 2*H*, Ar H), 7.01 (d, ^4^*J*_H,H_ = 1.6 Hz, 1*H*, Ar H), 6.95 (dd, ^3^*J*_H,H_ = 8.0 Hz, ^4^*J*_H,H_ = 1.6 Hz, 1*H*, Ar H), 6.73 (dd, ^3^*J*_H,H_ = 8.0 Hz, 1*H*, Ar H), 5.90 (s, 2*H*, O-CH_2_), 4.62 (bs, 2*H*, NH_2_), 4.46 (s, 2*H*, Ar-CH_2_-Ar), 4.20 (s, 2*H*, Ar-CH_2_-Ar), 3.89 (s, 3*H*, OCH_3_). ^13^C NMR (100 MHz, CDCl_3_): 167.0, 164.8, 151.4, 148.0, 147.8, 146.8, 145.6, 142.0, 140.1, 133.0, 130.2, 129.1, 129.0, 128.7, 128.6, 122.9, 122.7, 116.4, 110.0, 108.4, 101.1, 52.2, 42.0, 38.8. MS (ESI), *m*/*z* [M + H]^+^: 468.35. HRMS (ESI), *m*/*z* [M + H]^+^: calculated for C_26_H_22_N_5_O_4_^+^ 468.1667; found 468.1664. HPLC rt: 19.7 min.

#### 4-(3-(3-Amino-1*H*-1,2,4-triazol-5-yl)-4-(2-(benzo[*d*][1,3]dioxol-5-yl)acetamido)benzyl)benzoic acid (17)

To a solution of 15 (700 mg, 1.5 mmol, 1 equiv.) in 1,4-dioxane/water (3 : 1, v/v, 10 mL) was added LiOH (287 mg, 12 mmol, 8 equiv.) and the mixture was left stirring at room temperature overnight. The reaction was concentrated under reduced pressure, the residue was taken up in water and adjusted to pH 4 with 2 N HCl. The formed precipitate was filtered, washed with water and dried under vacuum to afford 17 as a white solid (590 mg, 86%); mp. = <260 °C with decomposition. ^1^H NMR (400 MHz, DMSO-*d*_6_): 7.97 (s, 1*H*, Ar H), 7.89 (d, ^3^*J*_H,H_ = 8.2 Hz, 2*H*, Ar H), 7.82 (d, ^3^*J*_H,H_ = 8.0 Hz, 1*H*, Ar H), 7.69 (d, ^3^*J*_H,H_ = 8.0 Hz, 1*H*, Ar H), 7.42 (d, ^3^*J*_H,H_ = 8.2 Hz, 2*H*, Ar H), 6.97 (s, 1*H*, Ar H), 6.82 (s, 2*H*, Ar H), 5.96 (s, 2*H*, O-CH_2_), 4.39 (s, 2*H*, Ar-CH_2_-Ar), 4.26 (s, 2*H*, Ar-CH_2_-Ar). ^13^C NMR (100 MHz, DMSO-*d*_6_): 167.8, 166.3, 150.8, 148.1, 147.8, 146.9, 146.4, 141.6, 140.9, 133.3, 129.8, 129.5, 128.6, 122.8, 122.7, 116.1, 110.3, 108.8, 101.5, 41.1, 38.5. MS (ESI), *m*/*z* [M + H]^+^: 472.26. HRMS (ESI), *m*/*z* [M + H]^+^: calculated for C_25_H_22_N_5_O_5_^+^ 472.1615; found 472.1620. HPLC rt: 14.22 min.

#### 4-(3-(3-Amino-1*H*-1,2,4-triazol-5-yl)-4-(2-(benzo[*d*][1,3]dioxol-5-yl)acetamido)benzyl)-*N*-hydroxybenzamide (18)

17 (100 mg, 0.21 mmol, 1 equiv.) and HOBt (34 mg, 0.25 mmol, 1.2 equiv.) were dissolved in dry DMF (4 mL) and DIPEA (0.09 mL, 0.52 mmol, 2.5 equiv.) was added. After 30 min, EDCI (48 mg, 0.25 mmol, 1.2 equiv.) and NH_2_OTMS (0.035 mL, 0.29 mmol, 1.4 equiv.) were added and the mixture was left stirring at room temperature overnight. The reaction mixture was then diluted with ethyl acetate (10 mL) and was washed with saturated aq. solution of NH_4_Cl (3 × 8 mL), saturated aq. solution of NaHCO_3_ (3 × 8 mL) and brine (3 × 8 mL). The organic layer was dried with anhydrous Na_2_SO_4_, filtered and concentrated under reduced pressure. The crude was purified *via* automatic flash column chromatography (reverse phase, water/acetonitrile gradient from 5% to 100%) to afford 18 as a white solid (43% yield, 44 mg); mp. = <264 °C with decomposition. ^1^H NMR (400 MHz, DMSO-*d*_6_): 12.34 (s, 1*H*, NH), 11.88 (bs, 1*H*, OH), 11.11 (s, 1*H*, NH), 8.94 (bs, 1*H*, NH), 8.40 (d, ^3^*J*_H,H_ = 8.6 Hz, 1*H*, Ar H), 7.84 (d, ^4^*J*_H,H_ = 2.2 Hz, 1*H*, Ar H), 7.66 (d, ^3^*J*_H,H_ = 8.6 Hz, 2*H*, Ar H), 7.28 (d, ^3^*J*_H,H_ = 8.6 Hz, 2*H*, Ar H), 7.19 (d, ^3^*J*_H,H_ = 8.2 Hz, 1*H*, Ar H), 6.93 (m, 1*H*, Ar H), 6.85 (m, 2*H*, Ar H), 6.32 (bs, 2*H*, NH_2_), 5.97 (s, 2*H*, O-CH_2_), 3.96 (s, 2*H*, Ar-CH_2_-Ar), 3.61 (s, 2*H*, Ar-CH_2_-Ar). ^13^C NMR (100 MHz, DMSO-*d*_6_): 169.0, 157.4, 156.3, 148.7, 147.2, 146.0, 144.6, 135.1, 134.9, 130.5, 129.3, 129.0, 128.5, 127.2, 127.0, 122.4, 119.4, 118.3, 109.6, 108.1, 100.8, 44.5, 40.2. MS (ESI), *m*/*z* [M + H]^+^: 487.26. HRMS (ESI), *m*/*z* [M + H]^+^: calculated for C_25_H_23_N_6_O_5_^+^ 487.1725; found 487.1721. HPLC rt: 11.77 min.

## Conflicts of interest

There are no conflicts to declare.

## Supplementary Material

RA-012-D2RA01753A-s001
