# Supplementary material for: Synthesis of potent and selective HDAC6 inhibitors led to unexpected opening of a quinazoline ring
Source: RSC Adv. 2022 Apr 13;12(18):11548–56. doi: 10.1039/d2ra01753a (PMC9006239; doi:10.1039/d2ra01753a)

Electronic Supplementary Material (ESI) for RSC Advances.
This journal is © The Royal Society of Chemistry 2022

Supplementary Materials

Synthesis of Potent and Selective HDAC6 Inhibitors led to Unexpected Opening of Quinazoline Ring

Davide Moi,[1,2,‡] Andrea Citarella,[1,2,‡] Davide Bonanni,[1] Luca Pinzi,[1] Daniele Passarella,[2] Alessandra Silvani,[2] Clelia Giannini[2] and Giulio Rastelli[1]*

[1] Department of Life Sciences, University of Modena and Reggio Emilia. Via Campi 103, 41125 Modena, Italy

[2] Department of Chemistry, University of Milan, Via Golgi 19, 20133 Milano, Italy

‡ Equally contributing authors

* Corresponding author: giulio.rastelli@unimore.it

Table of contents

	Pag.
Table S1	**S1**
Table S2	**S2-S6**
Figure S1-S3: Dose response curves	**S7**
^{1}H, ^{13}C NMR Spectra for all compounds	**S8-S19**
HPLC-MS for 11a and 18	**S20**

Table S1: *Chemical fragments including the benzohydroxamate, 1,3-benzodioxole and quinazoline substructures, identified among HDAC6 inhibitors. The number of compounds including the fragments and their prevalence ("Actives / Inactives rate") among the active and inactive classes are reported in the table.*

Fragment ID	*Fragment SMILES*	*Global Rank*	*Number of HDAC6 compounds*	*Number of active compounds*	*Number of inactive compounds*	*Actives / Inactives rate*	*Substructure Class*
Fragment 1	O=Cc1ccc(C(=O)NO)cc1	6	42	41	1	41.00	benzohydroxamate
Fragment 2	NCc1ccc(C(=O)NO)cc1	44	402	382	20	19.10	benzohydroxamate
Fragment 3	Cc1ccc(C(=O)NO)cc1	97	508	470	38	12.37	benzohydroxamate
Fragment 4	CNc1ccc(C(=O)NO)cc1	145	93	83	10	8.30	benzohydroxamate
Fragment 5	O=C(NO)c1ccccc1	167	694	611	83	7.36	benzohydroxamate
Fragment 6	Nc1ccc(C(=O)NO)cc1	173	99	87	12	7.25	benzohydroxamate
Fragment 7	O=C(NO)c1ccc(Cl)cc1	177	8	7	1	7.00	benzohydroxamate
Fragment 8	Nc1cc(C(=O)NO)ccc1Cl	183	8	7	1	7.00	benzohydroxamate
Fragment 9	O=Cc1cccc(C(=O)NO)c1	213	7	6	1	6.00	benzohydroxamate
Fragment 10	CCc1ccc(C(=O)NO)cc1	287	92	76	16	4.75	benzohydroxamate
Fragment 11	NCc1cccc(C(=O)NO)c1	421	23	18	5	3.60	benzohydroxamate
Fragment 12	c1ccc2c(c1)OCO2	230	33	28	5	5.60	1,3 benzodixole
Fragment 13	CCc1ccc2c(c1)OCO2	235	26	22	4	5.50	1,3 benzodixole
Fragment 14	Cc1ccc2c(c1)OCO2	238	32	27	5	5.40	1,3 benzodixole
Fragment 15	Cc1ncc2ccccc2n1	141	70	63	7	9.00	quinazoline
Fragment 16	c1ccc2ncncc2c1	491	151	115	36	3.19	quinazoline
Fragment 17	Cc1ncnc2ccccc12	509	4	3	1	3.00	quinazoline

Table S2: *Top-ranking similarities obtained for the derivatives 11a and 11b with respect to the HDAC6 ligands reported in ChEMBL, according to the MACCS and ECFP4 fingerprints. The similarity degree between the ligands is reported as a mean of the Tanimoto index. Only similarity values above commonly accepted thresholds are reported. The document reference ID of the ligands in ChEMBL are also reported.*

Compound ID	*ChEMBL ID*	*MACCSfp similarity score*	*ECFP4fp similarity score*	*Document ChEMBL ID*
**11a**	CHEMBL3655929	0.844	0.5	CHEMBL3639071
**11a**	CHEMBL3655997	0.831	0.459	CHEMBL3639071
**11a**	CHEMBL3601777	0.677	0.639	CHEMBL3600352
**11a**	CHEMBL4073021	0.633	0.606	CHEMBL4024792
**11a**	CHEMBL3314863	0.683	0.6	CHEMBL3351281
**11a**	CHEMBL3601776	0.734	0.564	CHEMBL3600352
**11a**	CHEMBL3918660	0.646	0.556	CHEMBL3886619
**11a**	CHEMBL3798183	0.645	0.556	CHEMBL3797085
**11a**	CHEMBL3893799	0.6	0.556	CHEMBL3886619
**11a**	CHEMBL3655914	0.656	0.545	CHEMBL3639071
**11a**	CHEMBL3652237	0.609	0.545	CHEMBL3639071
**11a**	CHEMBL3960564	0.612	0.541	CHEMBL3886619
**11a**	CHEMBL4466930	0.588	0.541	CHEMBL4385610
**11a**	CHEMBL4210907	0.631	0.537	CHEMBL4196108
**11a**	CHEMBL4073165	0.677	0.529	CHEMBL4024792
**11a**	CHEMBL4061807	0.661	0.529	CHEMBL4024792
**11a**	CHEMBL4079874	0.661	0.529	CHEMBL4024792
**11a**	CHEMBL4068310	0.645	0.529	CHEMBL4024792
**11a**	CHEMBL3909186	0.683	0.528	CHEMBL3886619
**11a**	CHEMBL3909854	0.683	0.528	CHEMBL3886619
**11a**	CHEMBL3927508	0.635	0.528	CHEMBL3886619
**11a**	CHEMBL3958794	0.603	0.528	CHEMBL4385610
**11a**	CHEMBL3979548	0.594	0.528	CHEMBL3886619
**11a**	CHEMBL3971436	0.582	0.528	CHEMBL4385610
**11a**	CHEMBL3798027	0.538	0.528	CHEMBL3797085
**11a**	CHEMBL3799183	0.538	0.528	CHEMBL3797085
**11a**	CHEMBL4160296	0.492	0.528	CHEMBL4130365
**11a**	CHEMBL4168213	0.422	0.528	CHEMBL4130365
**11a**	CHEMBL4080014	0.618	0.526	CHEMBL4033665
**11a**	CHEMBL2170177	0.615	0.526	CHEMBL4616727
**11a**	CHEMBL4225495	0.758	0.525	CHEMBL4219109
**11a**	CHEMBL3652239	0.645	0.515	CHEMBL3639071
**11a**	CHEMBL3655907	0.625	0.515	CHEMBL3639071
**11a**	CHEMBL3652226	0.615	0.515	CHEMBL3639071
**11a**	CHEMBL3652238	0.609	0.515	CHEMBL3639071
**11a**	CHEMBL4064437	0.661	0.514	CHEMBL4024792
**11a**	CHEMBL2170166	0.603	0.514	CHEMBL3639018
**11a**	CHEMBL3948680	0.594	0.514	CHEMBL3886619
**11a**	CHEMBL4280808	0.662	0.513	CHEMBL4261629
**11a**	CHEMBL4282039	0.603	0.513	CHEMBL4261629
**11a**	CHEMBL4537561	0.562	0.513	CHEMBL4422610
**11a**	CHEMBL4100664	0.556	0.513	CHEMBL3994606

**11a**	CHEMBL4069287	0.532	0.513	CHEMBL4033665
**11a**	CHEMBL4206021	0.627	0.512	CHEMBL4196108
**11a**	CHEMBL2425970	0.614	0.512	CHEMBL2424579
**11a**	CHEMBL2425971	0.614	0.512	CHEMBL2424579
**11a**	CHEMBL3217996	0.565	0.512	CHEMBL3217617
**11a**	CHEMBL3217999	0.565	0.512	CHEMBL3217617
**11a**	CHEMBL3217995	0.547	0.512	CHEMBL3217617
**11a**	CHEMBL3217998	0.547	0.512	CHEMBL3217617
**11a**	CHEMBL4228987	0.758	0.5	CHEMBL4219109
**11a**	CHEMBL1630117	0.754	0.5	CHEMBL1629443
**11a**	CHEMBL4529251	0.719	0.5	CHEMBL4414648
**11a**	CHEMBL4535144	0.719	0.5	CHEMBL4414648
**11a**	CHEMBL1630118	0.7	0.5	CHEMBL1629443
**11a**	CHEMBL4551484	0.688	0.5	CHEMBL4414648
**11a**	CHEMBL3314862	0.683	0.5	CHEMBL3351281
**11a**	CHEMBL4464235	0.672	0.5	CHEMBL4414648
**11a**	CHEMBL3652243	0.662	0.5	CHEMBL3639071
**11a**	CHEMBL4093718	0.661	0.5	CHEMBL4024792
**11a**	CHEMBL4556111	0.657	0.5	CHEMBL4354804
**11a**	CHEMBL3218000	0.651	0.5	CHEMBL3217617
**11a**	CHEMBL4531802	0.643	0.5	CHEMBL4385610
**11a**	CHEMBL3652225	0.639	0.5	CHEMBL3639071
**11a**	CHEMBL3217993	0.631	0.5	CHEMBL3217617
**11a**	CHEMBL4277089	0.631	0.5	CHEMBL4261629
**11a**	CHEMBL3926575	0.627	0.5	CHEMBL3886619
**11a**	CHEMBL3652231	0.615	0.5	CHEMBL3639071
**11a**	CHEMBL3652240	0.615	0.5	CHEMBL3639071
**11a**	CHEMBL2425952	0.614	0.5	CHEMBL2424579
**11a**	CHEMBL3952983	0.611	0.5	CHEMBL3886619
**11a**	CHEMBL4214369	0.609	0.5	CHEMBL4196108
**11a**	CHEMBL4475240	0.606	0.5	CHEMBL4385610
**11a**	CHEMBL4209463	0.603	0.5	CHEMBL4196108
**11a**	CHEMBL4560818	0.594	0.5	CHEMBL4414648
**11a**	CHEMBL2018304	0.592	0.5	CHEMBL2016572
**11a**	CHEMBL2018450	0.592	0.5	CHEMBL2016572
**11a**	CHEMBL4206537	0.591	0.5	CHEMBL4196108
**11a**	CHEMBL4438279	0.586	0.5	CHEMBL4385610
**11a**	CHEMBL4215954	0.576	0.5	CHEMBL4196108
**11a**	CHEMBL3898702	0.567	0.5	CHEMBL3886619
**11a**	CHEMBL4468860	0.565	0.5	CHEMBL4385610
**11a**	CHEMBL482094	0.448	0.5	CHEMBL1155020
**11a**	CHEMBL4441436	0.643	0.488	CHEMBL4330076
**11a**	CHEMBL4541796	0.636	0.488	CHEMBL4385610
**11a**	CHEMBL4218266	0.631	0.488	CHEMBL4196108
**11a**	CHEMBL4517827	0.629	0.488	CHEMBL4330076
**11a**	CHEMBL4464975	0.616	0.488	CHEMBL4330076
**11a**	CHEMBL4565933	0.616	0.488	CHEMBL4330076
**11a**	CHEMBL4442777	0.603	0.488	CHEMBL4330076
**11a**	CHEMBL4470654	0.603	0.488	CHEMBL4330076

**11a**	CHEMBL4579683	0.603	0.488	CHEMBL4330076
**11a**	CHEMBL3217994	0.569	0.488	CHEMBL3217617
**11a**	CHEMBL3217997	0.569	0.488	CHEMBL3217617
**11a**	CHEMBL3314869	0.625	0.487	CHEMBL3351281
**11a**	CHEMBL4470067	0.623	0.487	CHEMBL4385610
**11a**	CHEMBL4445881	0.58	0.487	CHEMBL4385610
**11a**	CHEMBL4066920	0.565	0.487	CHEMBL4385610
**11a**	CHEMBL4563723	0.725	0.486	CHEMBL4346670
**11a**	CHEMBL3655910	0.636	0.486	CHEMBL3639071
**11a**	CHEMBL3652242	0.625	0.486	CHEMBL3639071
**11b**	CHEMBL3655929	0.844	0.515	CHEMBL3639071
**11b**	CHEMBL3655997	0.831	0.472	CHEMBL3639071
**11b**	CHEMBL4225495	0.758	0.538	CHEMBL4219109
**11b**	CHEMBL4228987	0.758	0.513	CHEMBL4219109
**11b**	CHEMBL1630117	0.754	0.478	CHEMBL1629443
**11b**	CHEMBL3601776	0.734	0.538	CHEMBL3600352
**11b**	CHEMBL4563723	0.725	0.5	CHEMBL4346670
**11b**	CHEMBL4529251	0.719	0.475	CHEMBL4414648
**11b**	CHEMBL4535144	0.719	0.475	CHEMBL4414648
**11b**	CHEMBL1630118	0.7	0.478	CHEMBL1629443
**11b**	CHEMBL3314863	0.683	0.571	CHEMBL3351281
**11b**	CHEMBL3909186	0.683	0.5	CHEMBL3886619
**11b**	CHEMBL3909854	0.683	0.5	CHEMBL3886619
**11b**	CHEMBL3314862	0.683	0.475	CHEMBL3351281
**11b**	CHEMBL3601777	0.677	0.611	CHEMBL3600352
**11b**	CHEMBL4073165	0.677	0.5	CHEMBL4024792
**11b**	CHEMBL4439302	0.672	0.485	CHEMBL4414648
**11b**	CHEMBL3652229	0.667	0.485	CHEMBL3639071
**11b**	CHEMBL4280808	0.662	0.526	CHEMBL4261629
**11b**	CHEMBL3652243	0.662	0.515	CHEMBL3639071
**11b**	CHEMBL4061807	0.661	0.5	CHEMBL4024792
**11b**	CHEMBL4079874	0.661	0.5	CHEMBL4024792
**11b**	CHEMBL4064437	0.661	0.486	CHEMBL4024792
**11b**	CHEMBL4556111	0.657	0.474	CHEMBL4354804
**11b**	CHEMBL3655914	0.656	0.515	CHEMBL3639071
**11b**	CHEMBL3652228	0.656	0.5	CHEMBL3639071
**11b**	CHEMBL3218000	0.651	0.476	CHEMBL3217617
**11b**	CHEMBL3918660	0.646	0.528	CHEMBL3886619
**11b**	CHEMBL3652239	0.645	0.531	CHEMBL3639071
**11b**	CHEMBL3798183	0.645	0.528	CHEMBL3797085
**11b**	CHEMBL4068310	0.645	0.5	CHEMBL4024792
**11b**	CHEMBL4531802	0.643	0.474	CHEMBL4385610
**11b**	CHEMBL3655910	0.636	0.5	CHEMBL3639071
**11b**	CHEMBL3927508	0.635	0.5	CHEMBL3886619
**11b**	CHEMBL4073021	0.633	0.576	CHEMBL4024792
**11b**	CHEMBL4210907	0.631	0.512	CHEMBL4196108
**11b**	CHEMBL4218266	0.631	0.5	CHEMBL4196108
**11b**	CHEMBL3217993	0.631	0.475	CHEMBL3217617
**11b**	CHEMBL4277089	0.631	0.474	CHEMBL4261629

**11b**	CHEMBL3970950	0.629	0.485	CHEMBL3886619
**11b**	CHEMBL4206021	0.627	0.525	CHEMBL4196108
**11b**	CHEMBL3926575	0.627	0.474	CHEMBL3886619
**11b**	CHEMBL3655907	0.625	0.531	CHEMBL3639071
**11b**	CHEMBL3655909	0.625	0.486	CHEMBL3639071
**11b**	CHEMBL4080014	0.618	0.5	CHEMBL4033665
**11b**	CHEMBL4288702	0.618	0.475	CHEMBL4261629
**11b**	CHEMBL2170177	0.615	0.541	CHEMBL4616727
**11b**	CHEMBL3652226	0.615	0.531	CHEMBL3639071
**11b**	CHEMBL3652234	0.615	0.5	CHEMBL3639071
**11b**	CHEMBL3652236	0.615	0.5	CHEMBL3639071
**11b**	CHEMBL3655908	0.615	0.486	CHEMBL3639071
**11b**	CHEMBL2425970	0.614	0.488	CHEMBL2424579
**11b**	CHEMBL2425971	0.614	0.488	CHEMBL2424579
**11b**	CHEMBL2425952	0.614	0.476	CHEMBL2424579
**11b**	CHEMBL3960564	0.612	0.514	CHEMBL3886619
**11b**	CHEMBL4066975	0.612	0.475	CHEMBL4041521
**11b**	CHEMBL3952983	0.611	0.475	CHEMBL3886619
**11b**	CHEMBL3652238	0.609	0.531	CHEMBL3639071
**11b**	CHEMBL3652237	0.609	0.515	CHEMBL3639071
**11b**	CHEMBL4214369	0.609	0.512	CHEMBL4196108
**11b**	CHEMBL3652227	0.609	0.5	CHEMBL3639071
**11b**	CHEMBL3652230	0.609	0.5	CHEMBL3639071
**11b**	CHEMBL4475240	0.606	0.474	CHEMBL4385610
**11b**	CHEMBL4282039	0.603	0.526	CHEMBL4261629
**11b**	CHEMBL4209463	0.603	0.513	CHEMBL4196108
**11b**	CHEMBL3958794	0.603	0.5	CHEMBL4385610
**11b**	CHEMBL2170166	0.603	0.486	CHEMBL3639018
**11b**	CHEMBL3893799	0.6	0.528	CHEMBL3886619
**11b**	CHEMBL4093691	0.6	0.476	CHEMBL4041521
**11b**	CHEMBL4560818	0.594	0.515	CHEMBL4414648
**11b**	CHEMBL3979548	0.594	0.5	CHEMBL3886619
**11b**	CHEMBL3948680	0.594	0.486	CHEMBL3886619
**11b**	CHEMBL2018304	0.592	0.474	CHEMBL2016572
**11b**	CHEMBL2018450	0.592	0.474	CHEMBL2016572
**11b**	CHEMBL4206537	0.591	0.513	CHEMBL4196108
**11b**	CHEMBL4466930	0.588	0.514	CHEMBL4385610
**11b**	CHEMBL4438279	0.586	0.474	CHEMBL4385610
**11b**	CHEMBL2018295	0.583	0.488	CHEMBL2016572
**11b**	CHEMBL2018296	0.583	0.488	CHEMBL2016572
**11b**	CHEMBL3971436	0.582	0.5	CHEMBL4385610
**11b**	CHEMBL2018300	0.581	0.488	CHEMBL2016572
**11b**	CHEMBL2018301	0.581	0.488	CHEMBL2016572
**11b**	CHEMBL4215954	0.576	0.513	CHEMBL4196108
**11b**	CHEMBL4103801	0.574	0.474	CHEMBL4033665
**11b**	CHEMBL3217996	0.565	0.488	CHEMBL3217617
**11b**	CHEMBL3217999	0.565	0.488	CHEMBL3217617
**11b**	CHEMBL4468860	0.565	0.474	CHEMBL4385610
**11b**	CHEMBL4537561	0.562	0.526	CHEMBL4422610

**11b**	CHEMBL4100664	0.556	0.487	CHEMBL3994606
**11b**	CHEMBL3976023	0.556	0.485	CHEMBL3886619
**11b**	CHEMBL3217995	0.547	0.488	CHEMBL3217617
**11b**	CHEMBL3217998	0.547	0.488	CHEMBL3217617
**11b**	CHEMBL3798027	0.538	0.5	CHEMBL3797085
**11b**	CHEMBL3799183	0.538	0.5	CHEMBL3797085
**11b**	CHEMBL4069287	0.532	0.487	CHEMBL4033665
**11b**	CHEMBL4531108	0.531	0.488	CHEMBL4422610
**11b**	CHEMBL4283105	0.525	0.488	CHEMBL4422610
**11b**	CHEMBL4160296	0.492	0.5	CHEMBL4130365
**11b**	CHEMBL482094	0.448	0.474	CHEMBL1155020
**11b**	CHEMBL4168213	0.422	0.5	CHEMBL4130365

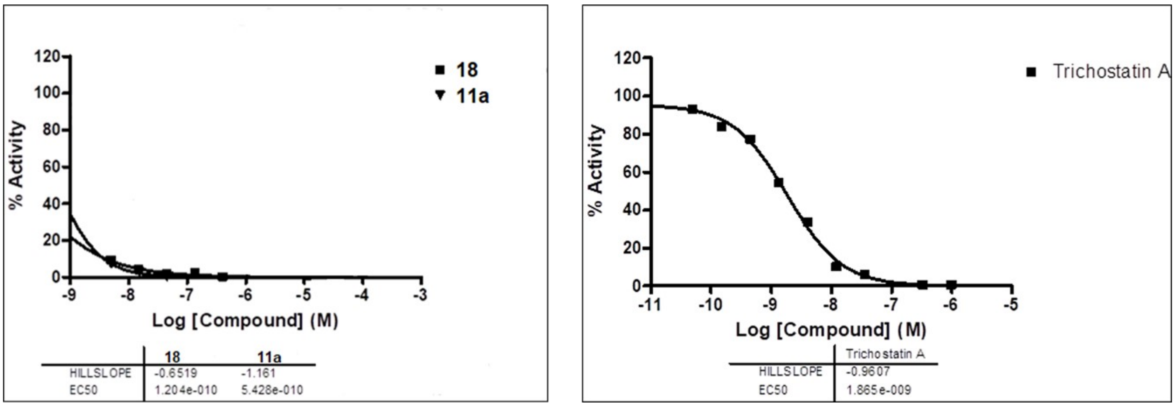

Figure S1. Dose response curves of **11a** and **18** tested against **HDAC6**. All compounds were tested in singlet 10-dose IC_{50} mode with 3-fold serial dilution starting at 100 µM**.** HDAC reference compound Trichostatin A (TSA) was tested in a 10-dose IC_{50} with 3-fold serial dilution starting at 1 µM. IC_{50} values were calculated using the GraphPad Prism4 program based on a sigmoidal dose-response equation.

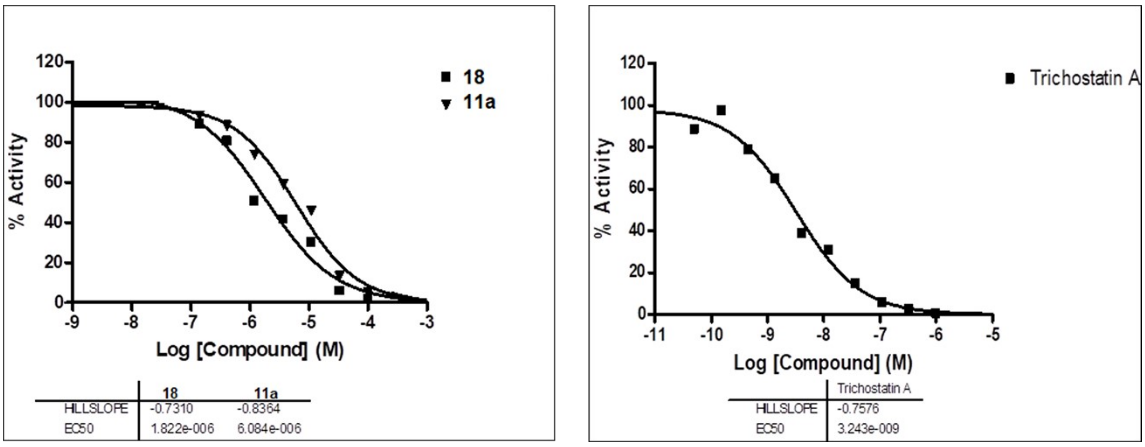

Figure S2. Dose response curves of **11a** and **18** tested against **HDAC1**. All compounds were tested in singlet 10-dose IC_{50} mode with 3-fold serial dilution starting at 100 µM. HDAC reference compound Trichostatin A (TSA) was tested in a 10-dose IC_{50} with 3-fold serial dilution starting at 1 µM. IC_{50} values were calculated using the GraphPad Prism4 program based on a sigmoidal dose-response equation.

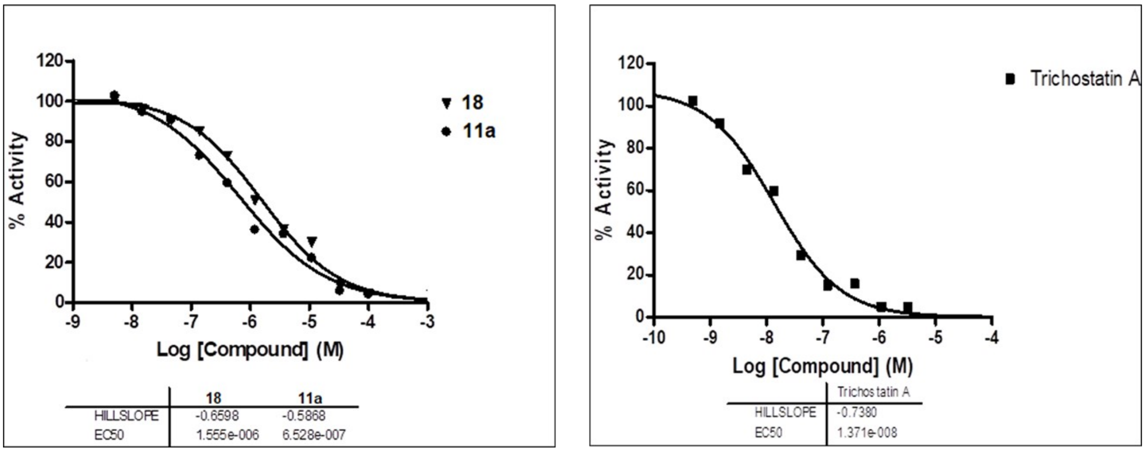

Figure S3. Dose response curves of **11a** and **18** tested against **HDAC8**. All compounds were tested in singlet 10-dose IC_{50} mode with 3-fold serial dilution starting at 100 µM. HDAC reference compound Trichostatin A (TSA) was tested in a 10-dose IC_{50} with 3-fold serial dilution starting at 1 µM. IC_{50} values were calculated using the GraphPad Prism4 program based on a sigmoidal dose-response equation.

^{1}H, ^{13}C NMR Spectra for all compounds

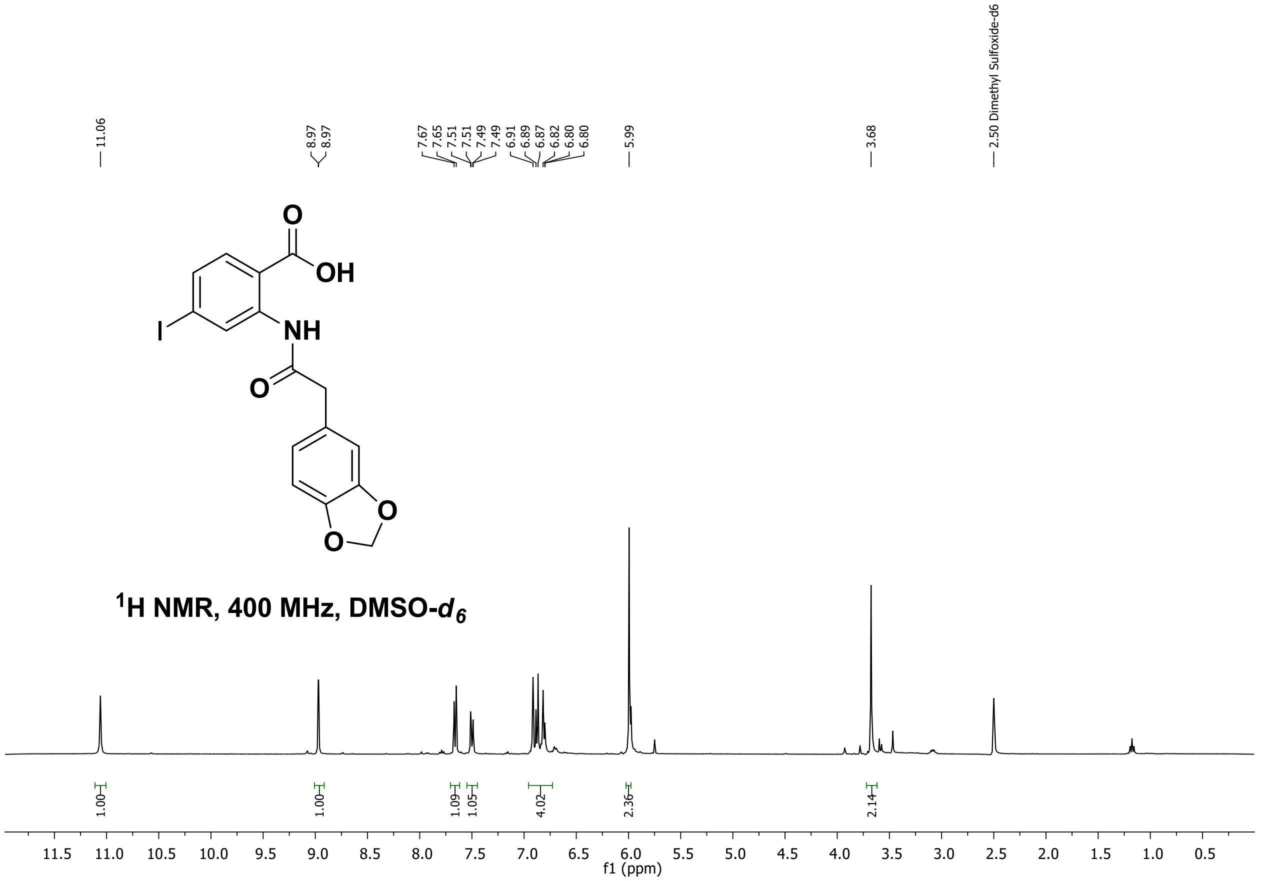

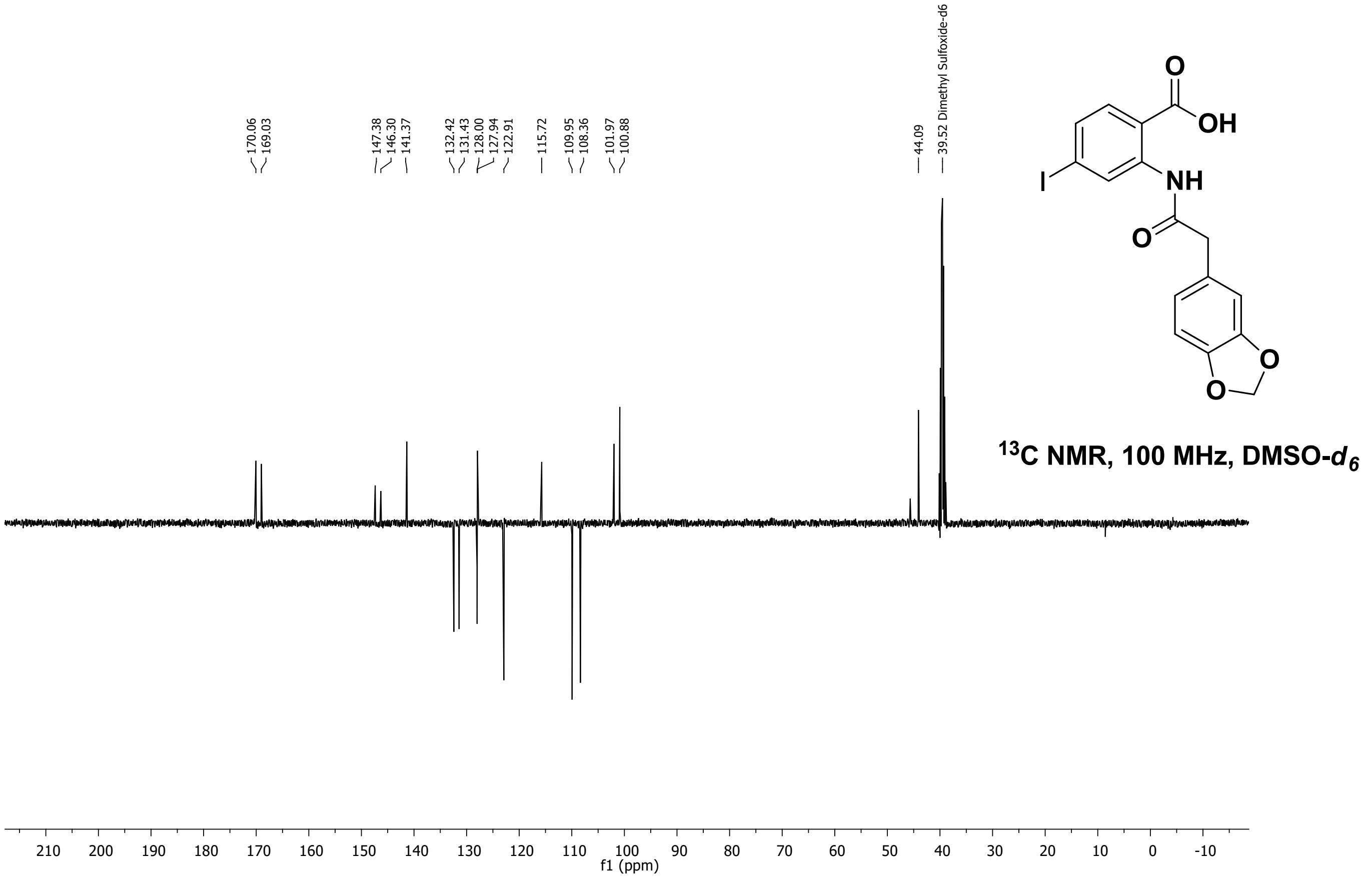

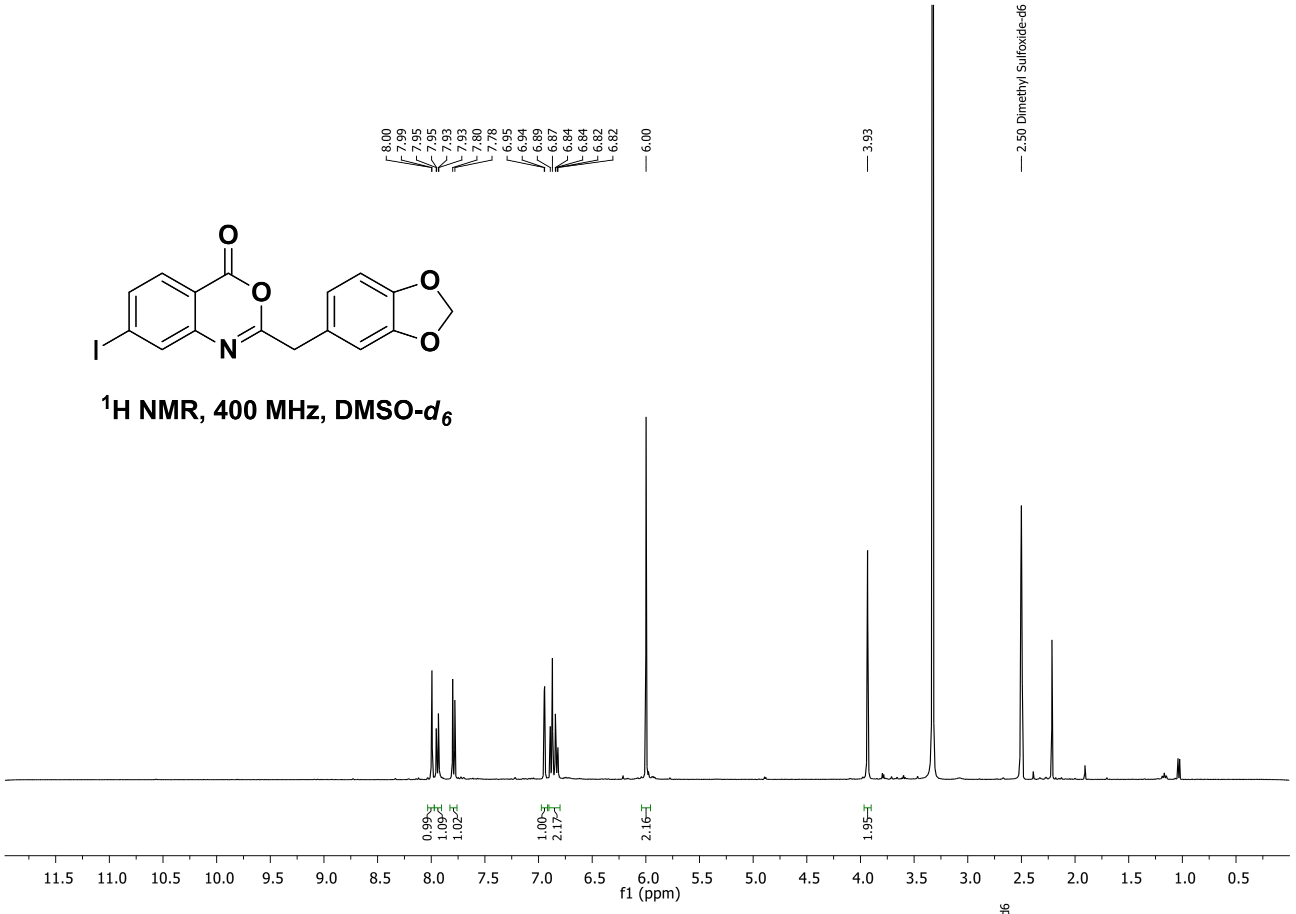
8.00
7.99
7.95
7.95
7.93
7.93
7.93
7.80
7.78
6.95
6.94
6.89
6.87
6.84
6.84
6.82
6.82
6.00
3.93
2.50 Dimethyl Sulfoxide-d6
^{1}H NMR, 400 MHz, DMSO-d_6
0.99
1.09
1.02
1.00
2.17
2.16
1.95
11.5 11.0 10.5 10.0 9.5 9.0 8.5 8.0 7.5 7.0 6.5 6.0 5.5 5.0 4.5 4.0 3.5 3.0 2.5 2.0 1.5 1.0 0.5
f1 (ppm)

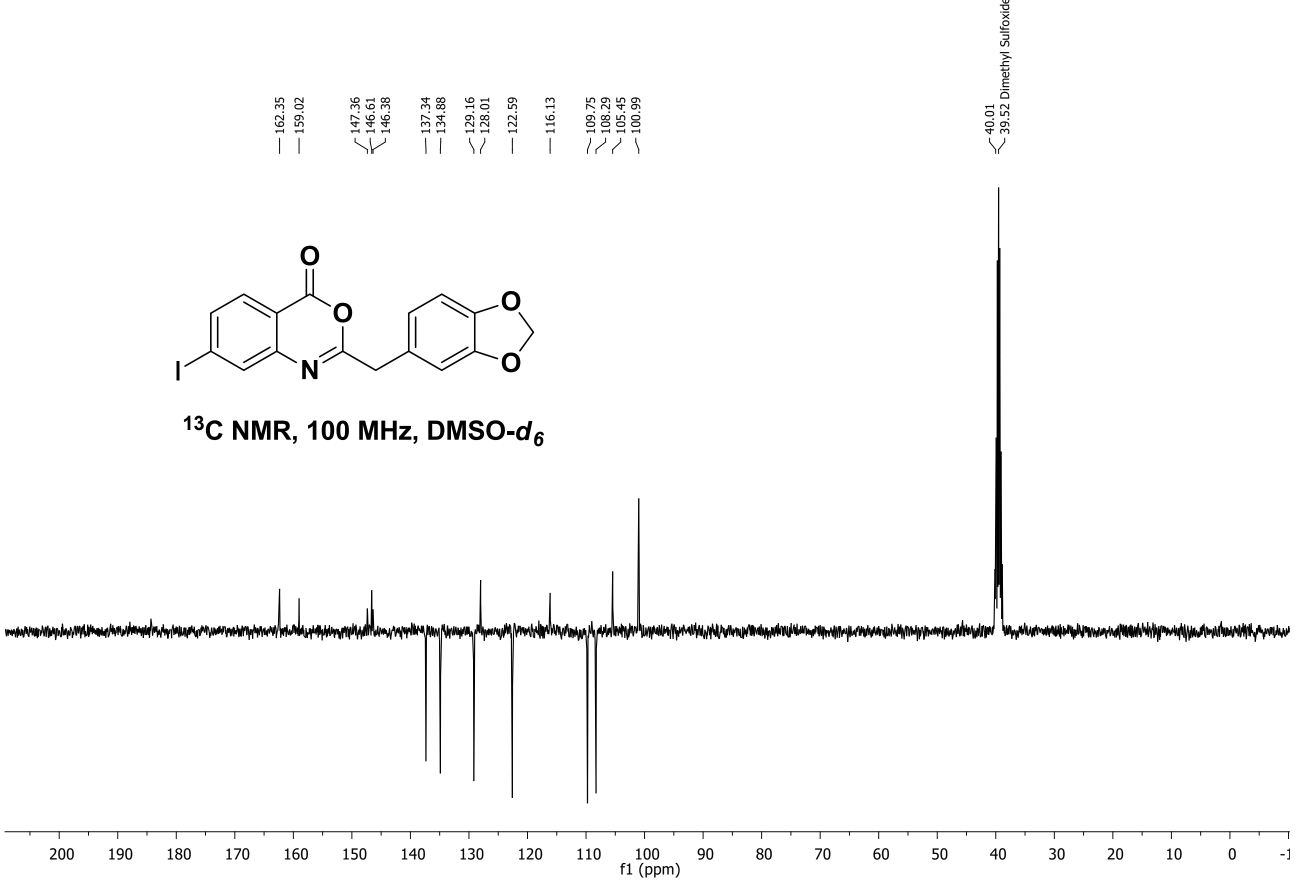
162.35
159.02
147.36
146.61
146.38
137.34
134.88
129.16
128.01
122.59
116.13
109.75
108.29
105.45
100.99
40.01
39.52 Dimethyl Sulfoxide-d6
^{13}C NMR, 100 MHz, DMSO-d_6
200 190 180 170 160 150 140 130 120 110 100 90 80 70 60 50 40 30 20 10 0
f1 (ppm)

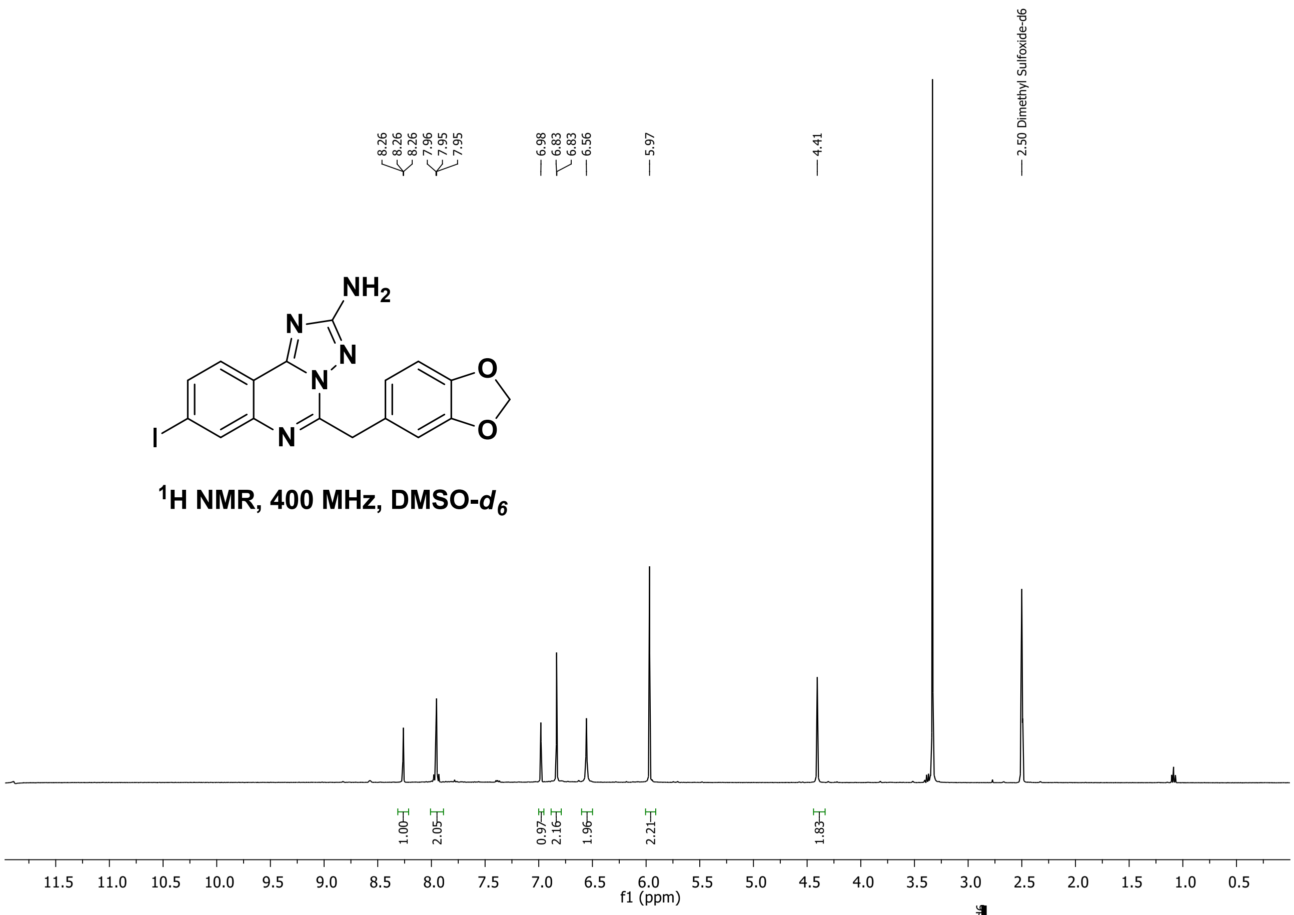
8.26
8.26
8.26
7.96
7.95
7.95
6.98
6.83
6.83
6.56
5.97
4.41
2.50 Dimethyl Sulfoxide-d6
NH2
N
N
N
N
O
O
I
1H NMR, 400 MHz, DMSO-d6
1.00
2.05
0.97
2.16
1.96
2.21
1.83
11.5 11.0 10.5 10.0 9.5 9.0 8.5 8.0 7.5 7.0 6.5 6.0 5.5 5.0 4.5 4.0 3.5 3.0 2.5 2.0 1.5 1.0 0.5
f1 (ppm)

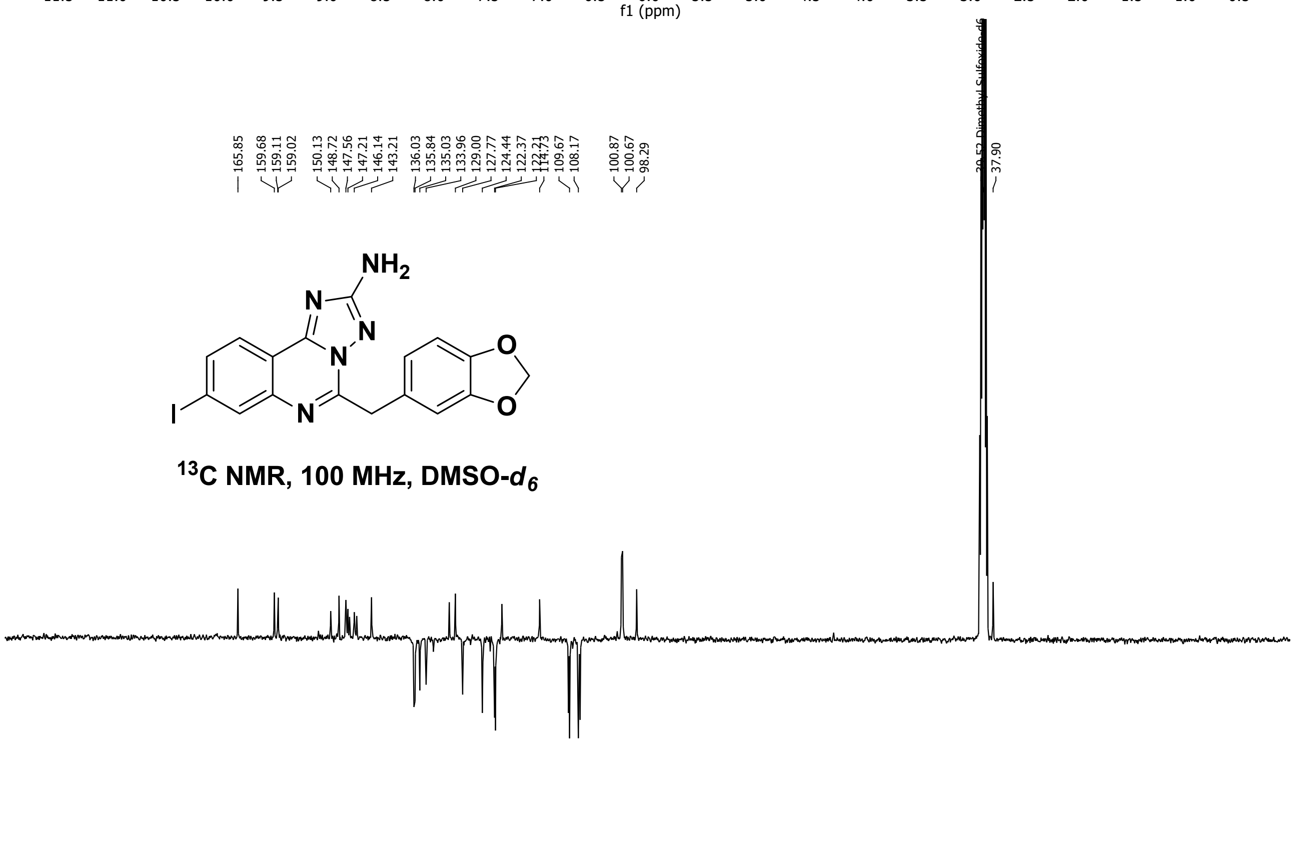
165.85
159.68
159.11
159.02
150.13
148.72
147.56
147.21
146.14
143.21
136.03
135.84
135.03
133.96
129.00
127.77
124.44
122.37
122.21
114.73
109.67
108.17
100.87
100.67
98.29
37.90
NH2
N
N
N
N
O
O
I
13C NMR, 100 MHz, DMSO-d6
200 190 180 170 160 150 140 130 120 110 100 90 80 70 60 50 40 30 20 10 0 -10
f1 (ppm)

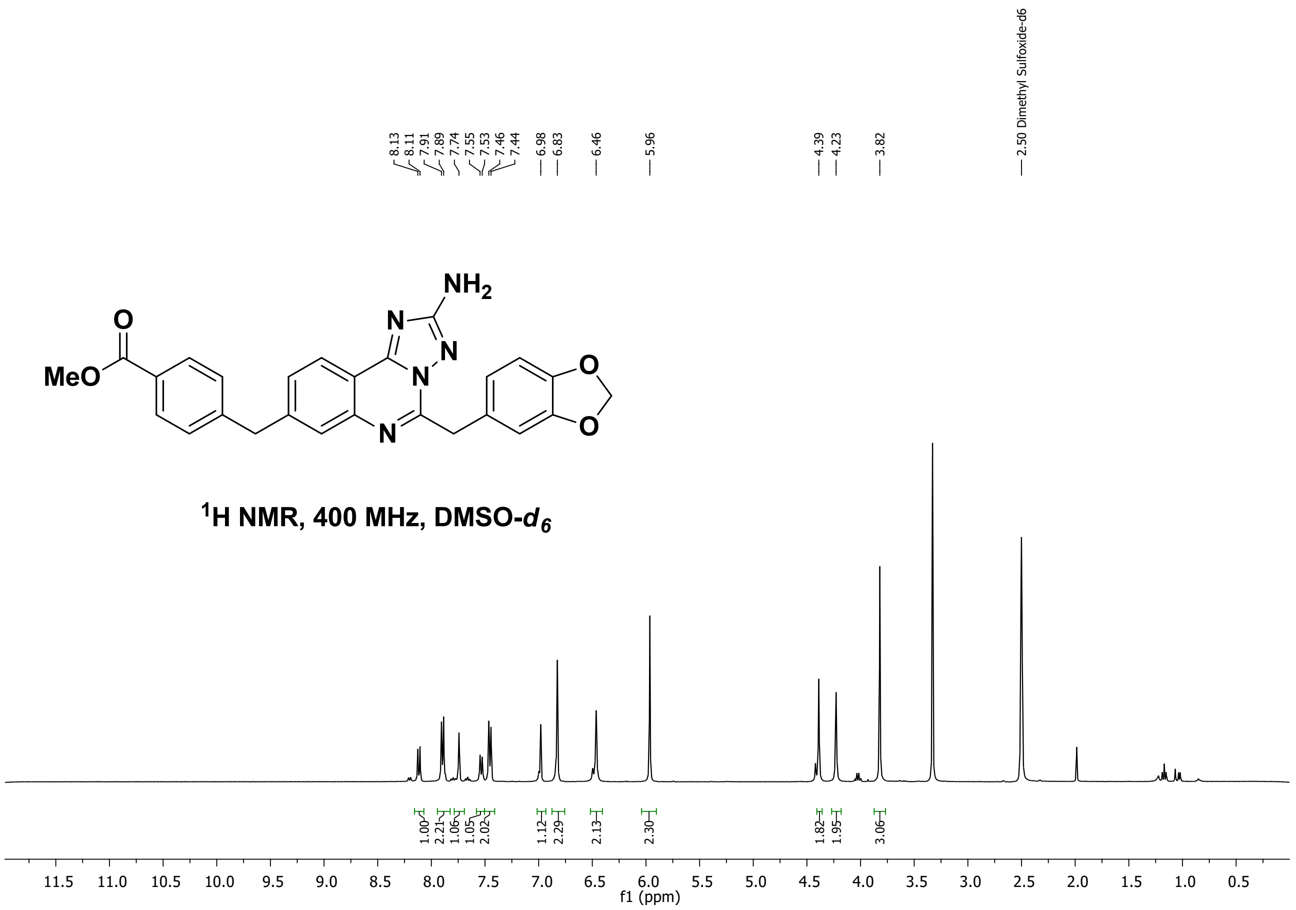
8.13
8.11
7.91
7.89
7.74
7.55
7.53
7.46
7.44
6.98
6.83
6.46
5.96
4.39
4.23
3.82
2.50 Dimethyl Sulfoxide-d6
NH2
O
N
N
N
MeO
O
O
N
1H NMR, 400 MHz, DMSO-d6
1.00
2.21
1.06
1.05
2.02
1.12
2.29
2.13
2.30
1.82
1.95
3.06
11.5 11.0 10.5 10.0 9.5 9.0 8.5 8.0 7.5 7.0 6.5 6.0 5.5 5.0 4.5 4.0 3.5 3.0 2.5 2.0 1.5 1.0 0.5
f1 (ppm)

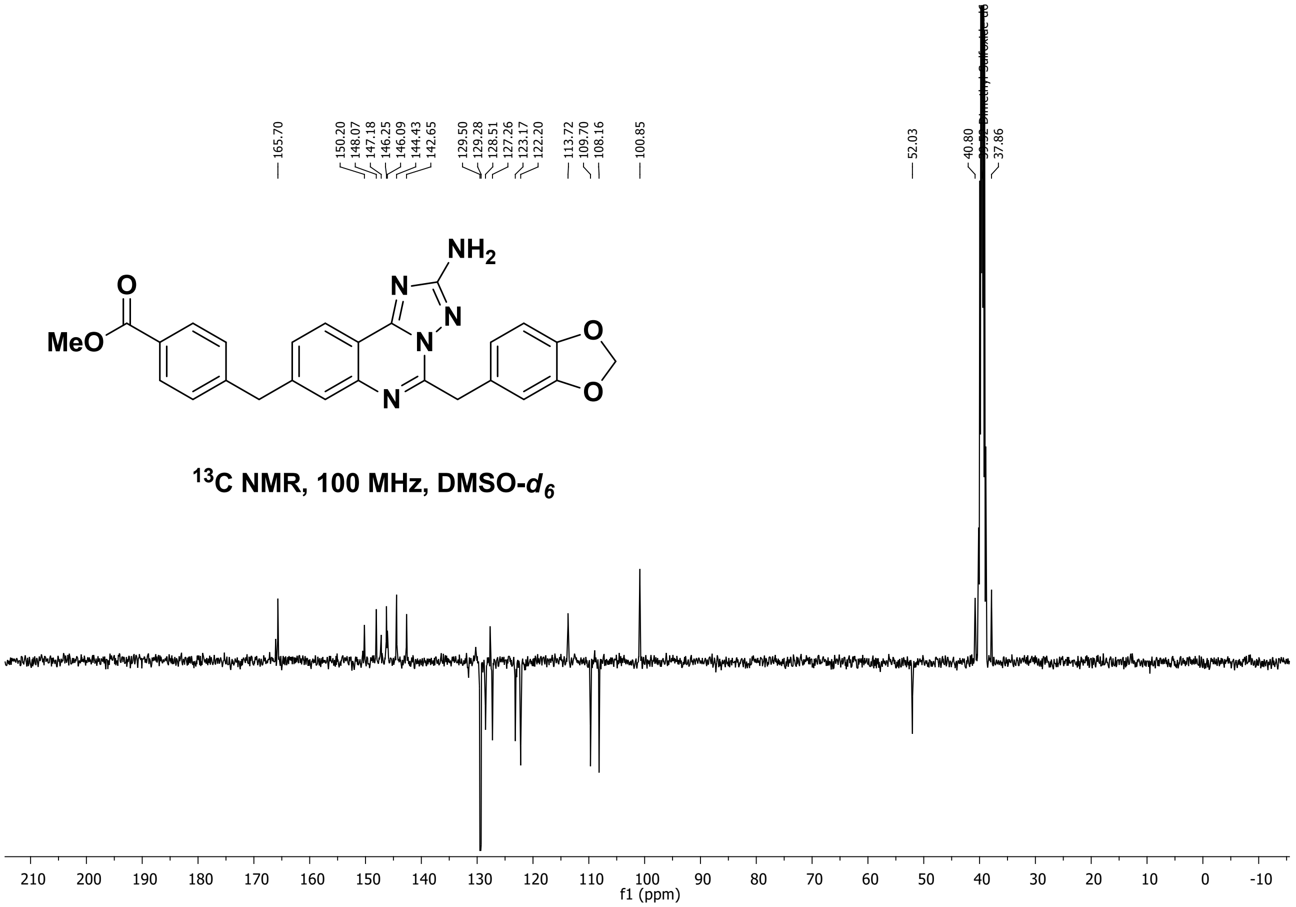
165.70
150.20
148.07
147.18
146.25
146.09
144.43
142.65
129.50
129.28
128.51
127.26
123.17
122.20
113.72
109.70
108.16
100.85
52.03
40.80
37.86
NH2
O
N
N
N
MeO
O
O
N
13C NMR, 100 MHz, DMSO-d6
210 200 190 180 170 160 150 140 130 120 110 100 90 80 70 60 50 40 30 20 10 0 -10
f1 (ppm)

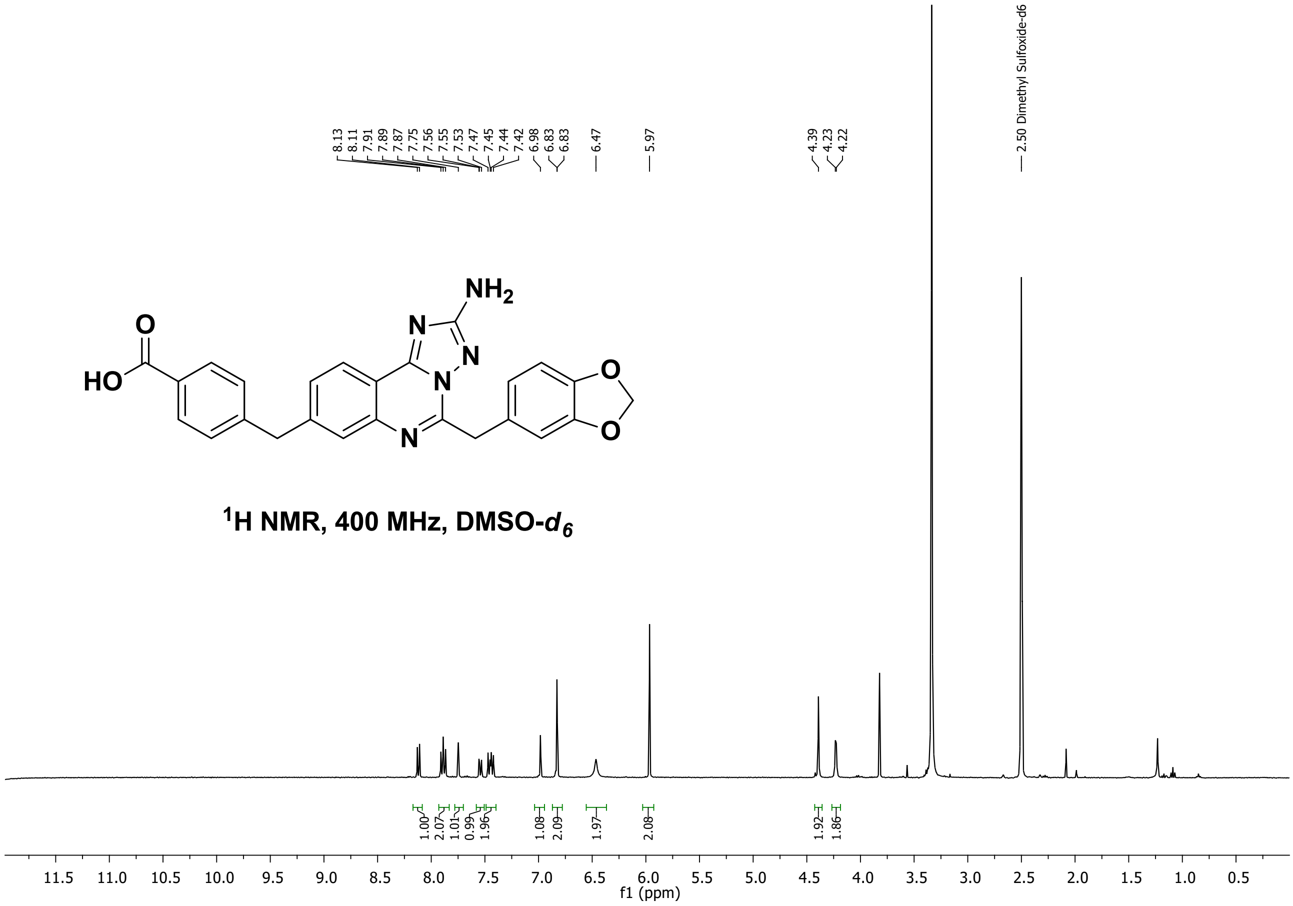
8.13
8.11
7.91
7.89
7.87
7.75
7.56
7.55
7.53
7.47
7.45
7.44
7.42
6.98
6.83
6.83
6.47
5.97
4.39
4.23
4.22
2.50 Dimethyl Sulfoxide-d6
NH2
O
N
N
HO
N
O
N
O
1H NMR, 400 MHz, DMSO-d6
1.00
2.07
1.01
0.99
1.96
1.08
2.09
1.97
2.08
1.92
1.86
11.5 11.0 10.5 10.0 9.5 9.0 8.5 8.0 7.5 7.0 6.5 6.0 5.5 5.0 4.5 4.0 3.5 3.0 2.5 2.0 1.5 1.0 0.5
f1 (ppm)

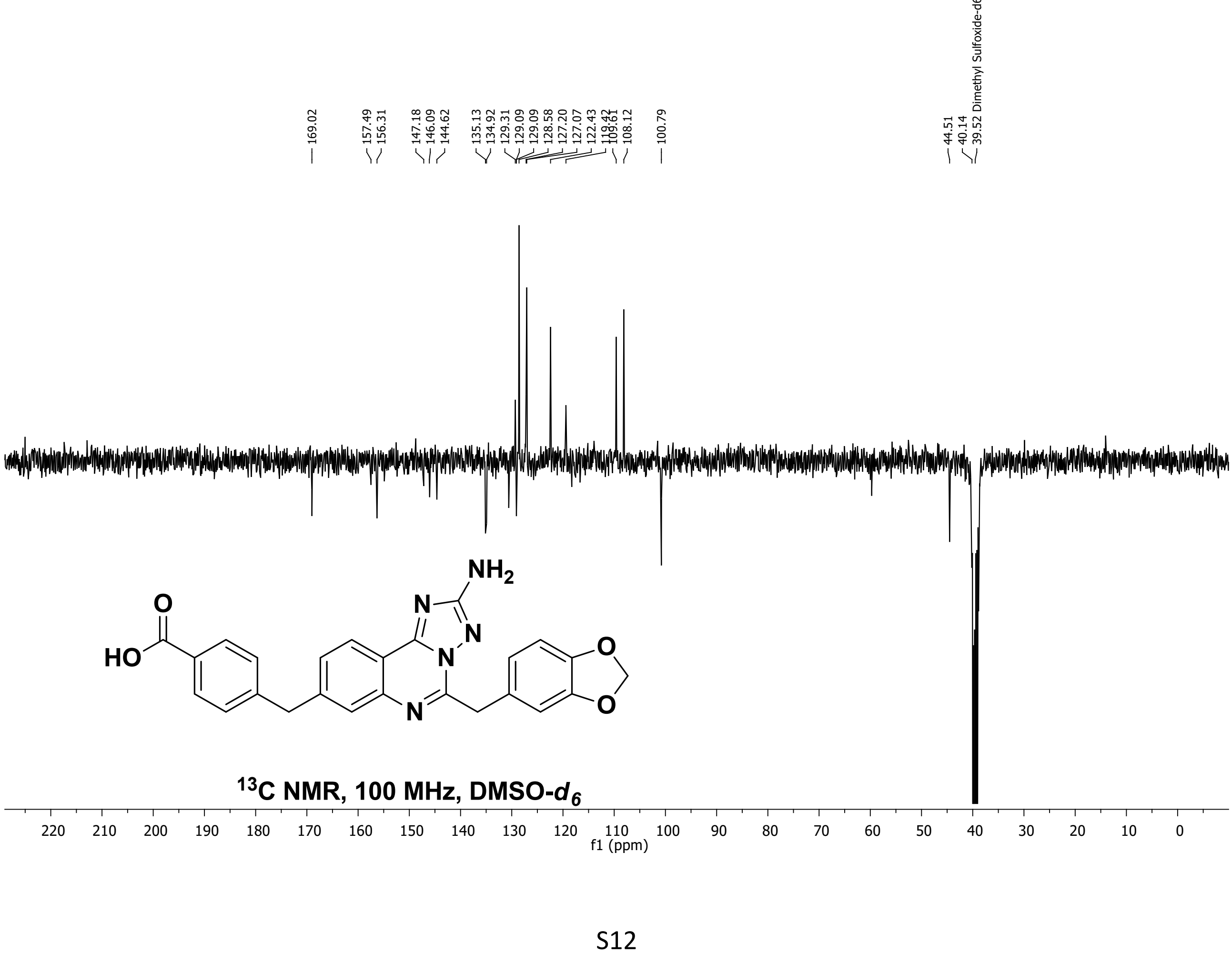
169.02
157.49
156.31
147.18
146.09
144.62
135.13
134.92
129.31
129.09
129.09
128.58
127.20
127.07
122.43
[illegible]
[illegible]
108.12
100.79
44.51
40.14
39.52 Dimethyl Sulfoxide-d6
NH2
O
N
N
HO
N
O
N
O
13C NMR, 100 MHz, DMSO-d6
220 210 200 190 180 170 160 150 140 130 120 110 100 90 80 70 60 50 40 30 20 10 0
f1 (ppm)

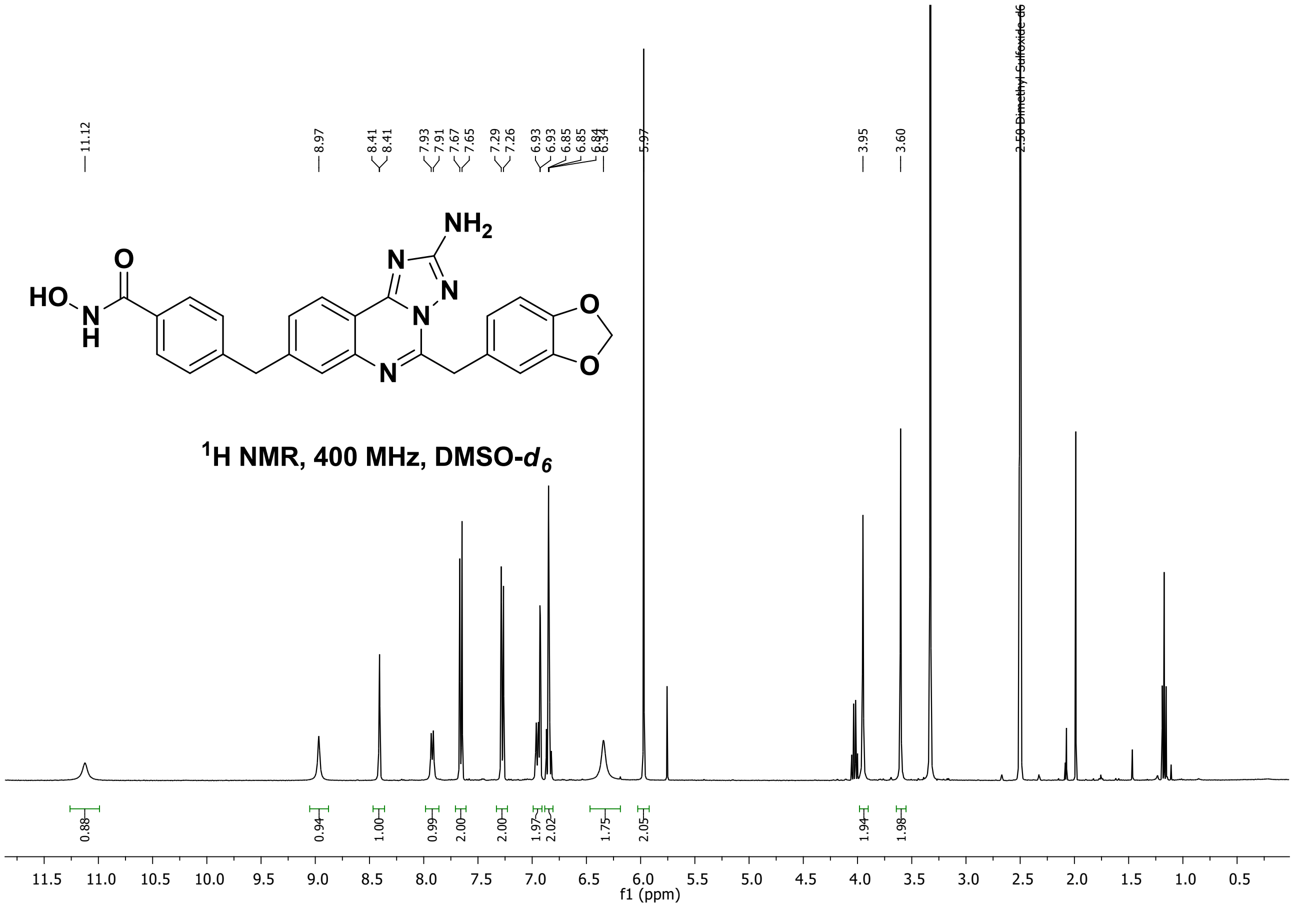

11.12
8.97
8.41
8.41
7.93
7.91
7.67
7.65
7.29
7.26
6.93
6.93
6.85
6.85
6.84
6.34
5.97
3.95
3.60
2.50 Dimethyl Sulfoxide-d6
NH2
O
N
HO
N
N
N
H
N
O
N
O
1H NMR, 400 MHz, DMSO-d6
0.88
0.94
1.00
0.99
2.00
2.00
1.97
2.02
1.75
2.05
1.94
1.98
f1 (ppm)

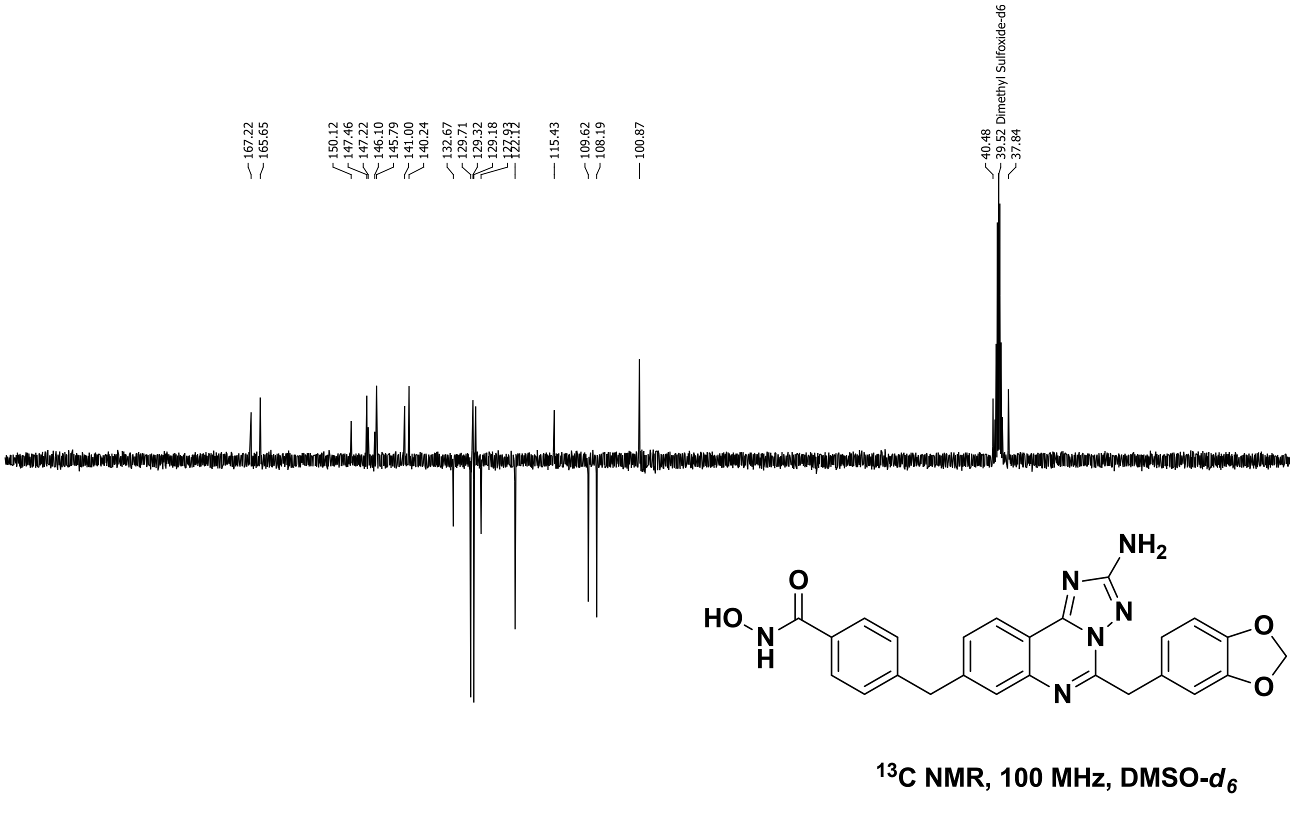

Dimethyl Sulfoxide-d6
167.22
165.65
150.12
147.46
147.22
146.10
145.79
141.00
140.24
132.67
129.71
129.32
129.18
127.93
122.12
115.43
109.62
108.19
100.87
40.48
39.52 Dimethyl Sulfoxide-d6
37.84
NH2
O
N
HO
N
N
N
H
N
O
N
O
13C NMR, 100 MHz, DMSO-d6
f1 (ppm)

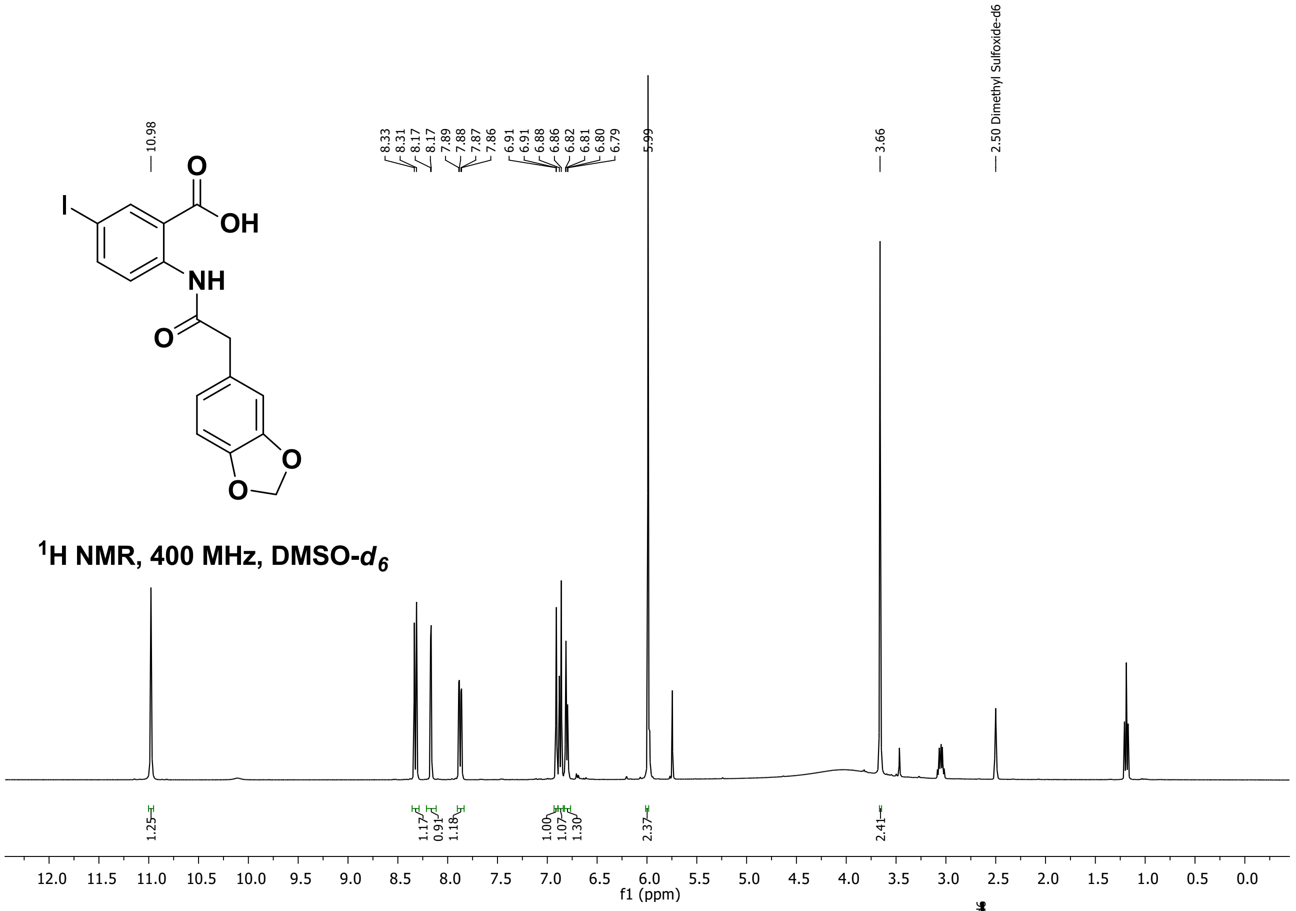
^{1}H NMR, 400 MHz, DMSO-d_6
10.98
8.33 8.31 8.17 8.17 7.89 7.88 7.87 7.86 6.91 6.91 6.88 6.86 6.82 6.81 6.80 6.79
5.99
3.66
2.50 Dimethyl Sulfoxide-d6
1.25 1.17 0.91 1.18 1.00 1.07 1.30 2.37 2.41
f1 (ppm)

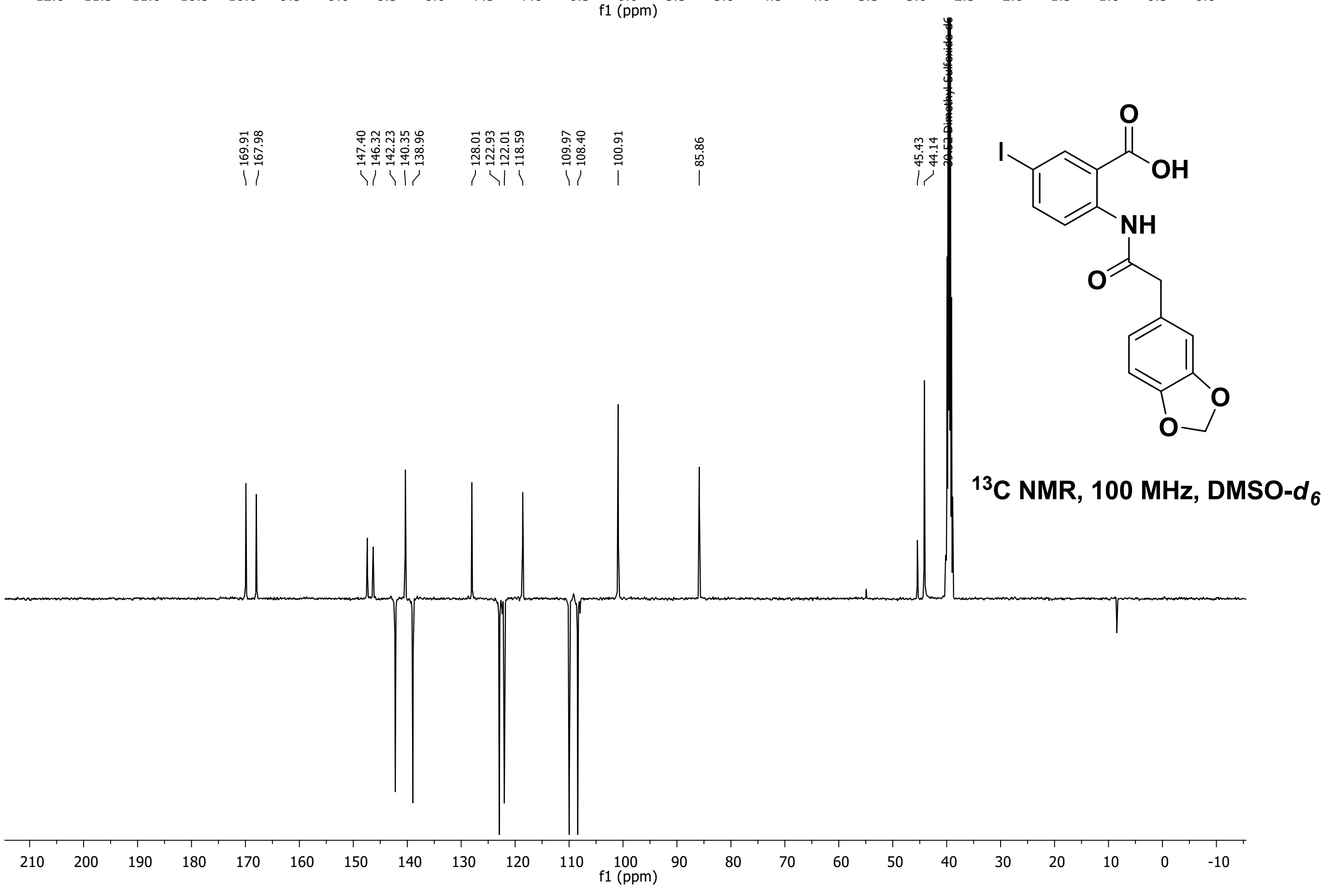
^{13}C NMR, 100 MHz, DMSO-d_6
169.91 167.98
147.40 146.32 142.23 140.35 138.96
128.01 122.93 122.01 118.59
109.97 108.40
100.91
85.86
45.43 44.14
f1 (ppm)

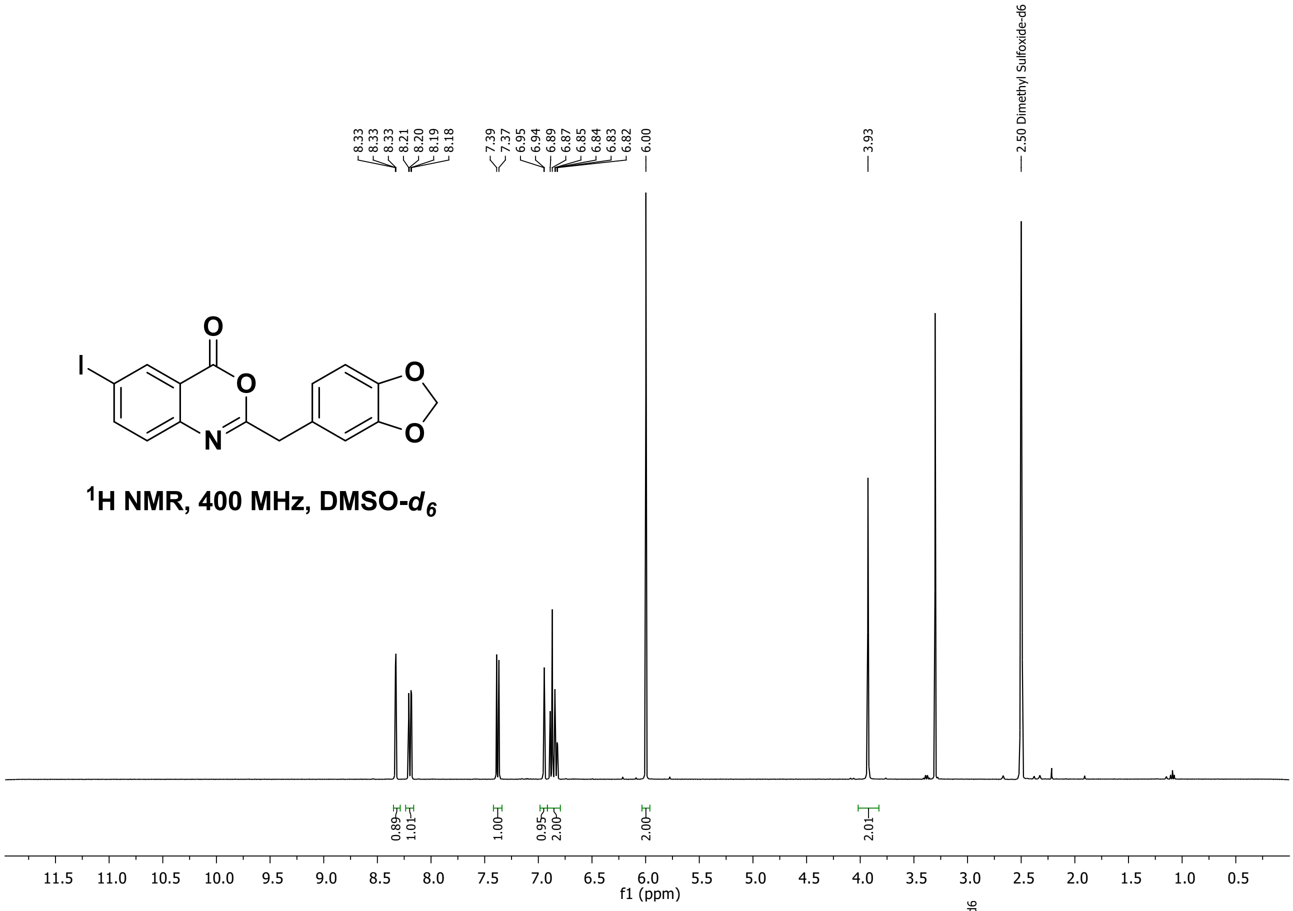
8.33
8.33
8.33
8.21
8.20
8.19
8.18
7.39
7.37
6.95
6.94
6.89
6.87
6.85
6.84
6.83
6.82
6.00
3.93
2.50 Dimethyl Sulfoxide-d6
O
I
O
N
O
O
1H NMR, 400 MHz, DMSO-d6
0.89
1.01
1.00
0.95
2.00
2.00
2.01
11.5
11.0
10.5
10.0
9.5
9.0
8.5
8.0
7.5
7.0
6.5
6.0
5.5
5.0
4.5
4.0
3.5
3.0
2.5
2.0
1.5
1.0
0.5
f1 (ppm)

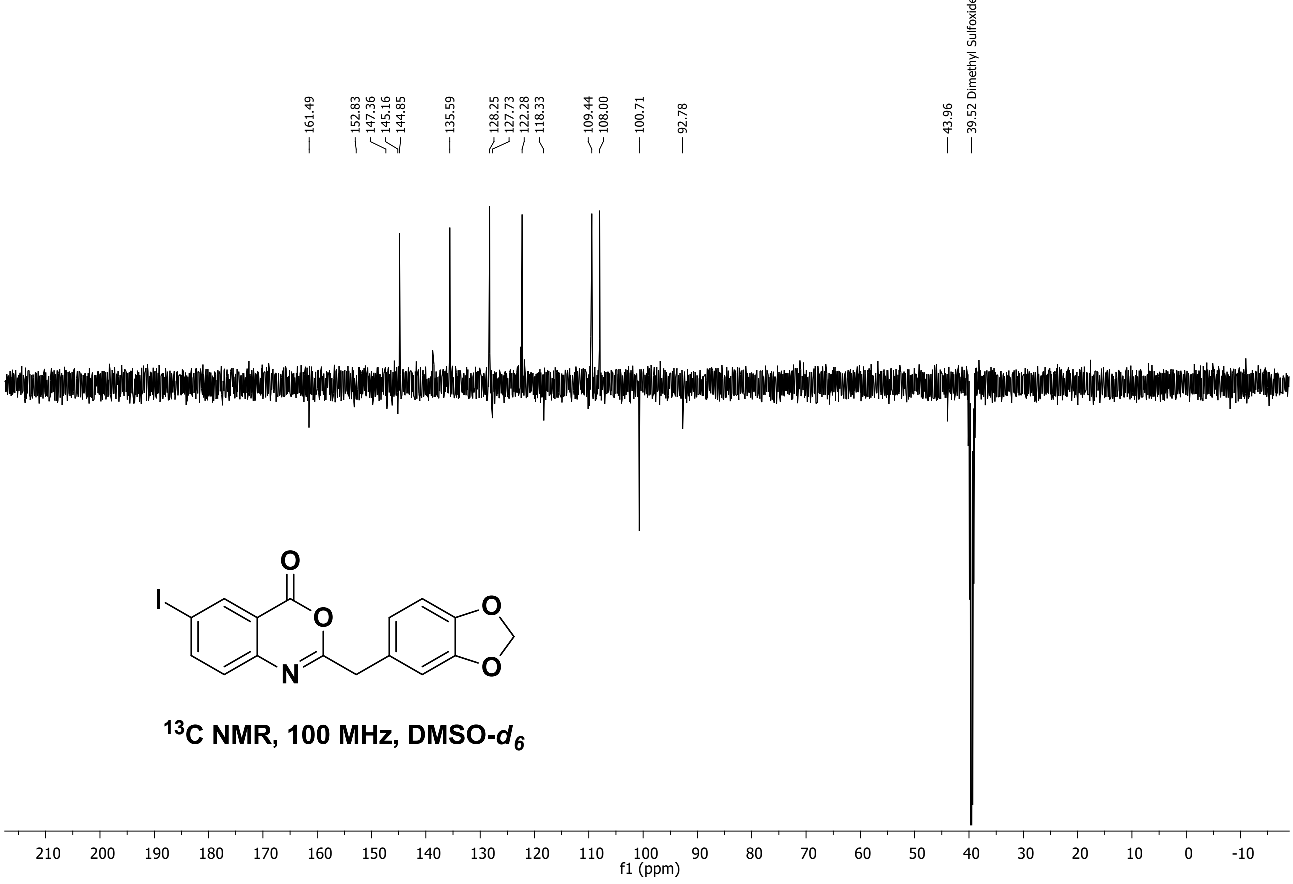
161.49
152.83
147.36
145.16
144.85
135.59
128.25
127.73
122.28
118.33
109.44
108.00
100.71
92.78
43.96
39.52 Dimethyl Sulfoxide-d6
O
I
O
N
O
O
13C NMR, 100 MHz, DMSO-d6
210
200
190
180
170
160
150
140
130
120
110
100
90
80
70
60
50
40
30
20
10
0
-10
f1 (ppm)

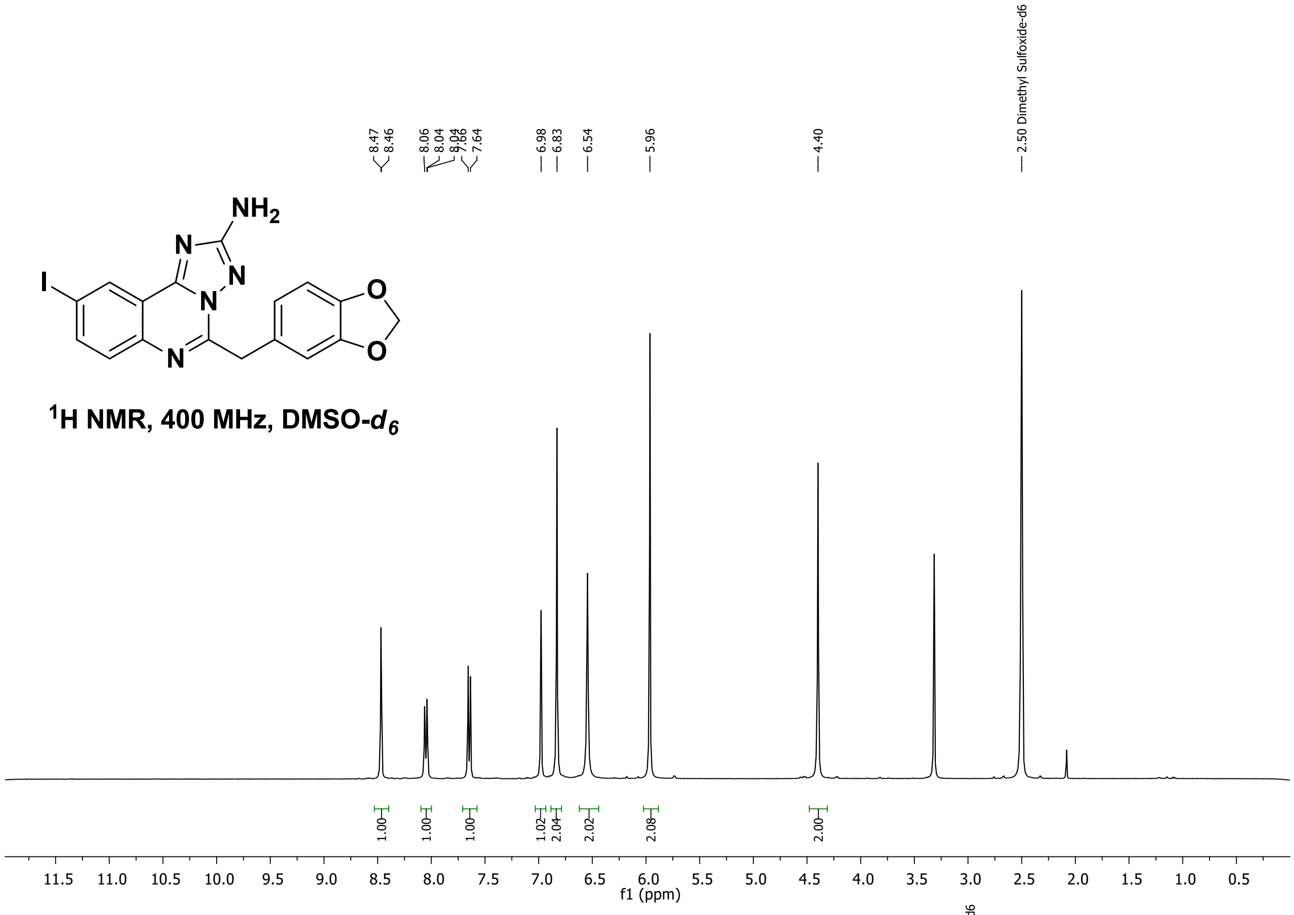

8.47
8.46
8.06
8.04
8.04
7.66
7.64
6.98
6.83
6.54
5.96
4.40
2.50 Dimethyl Sulfoxide-d6
NH2
N
N
N
I
O
O
N
1H NMR, 400 MHz, DMSO-d6
1.00
1.00
1.00
1.02
2.04
2.02
2.08
2.00
11.5 11.0 10.5 10.0 9.5 9.0 8.5 8.0 7.5 7.0 6.5 6.0 5.5 5.0 4.5 4.0 3.5 3.0 2.5 2.0 1.5 1.0 0.5
f1 (ppm)

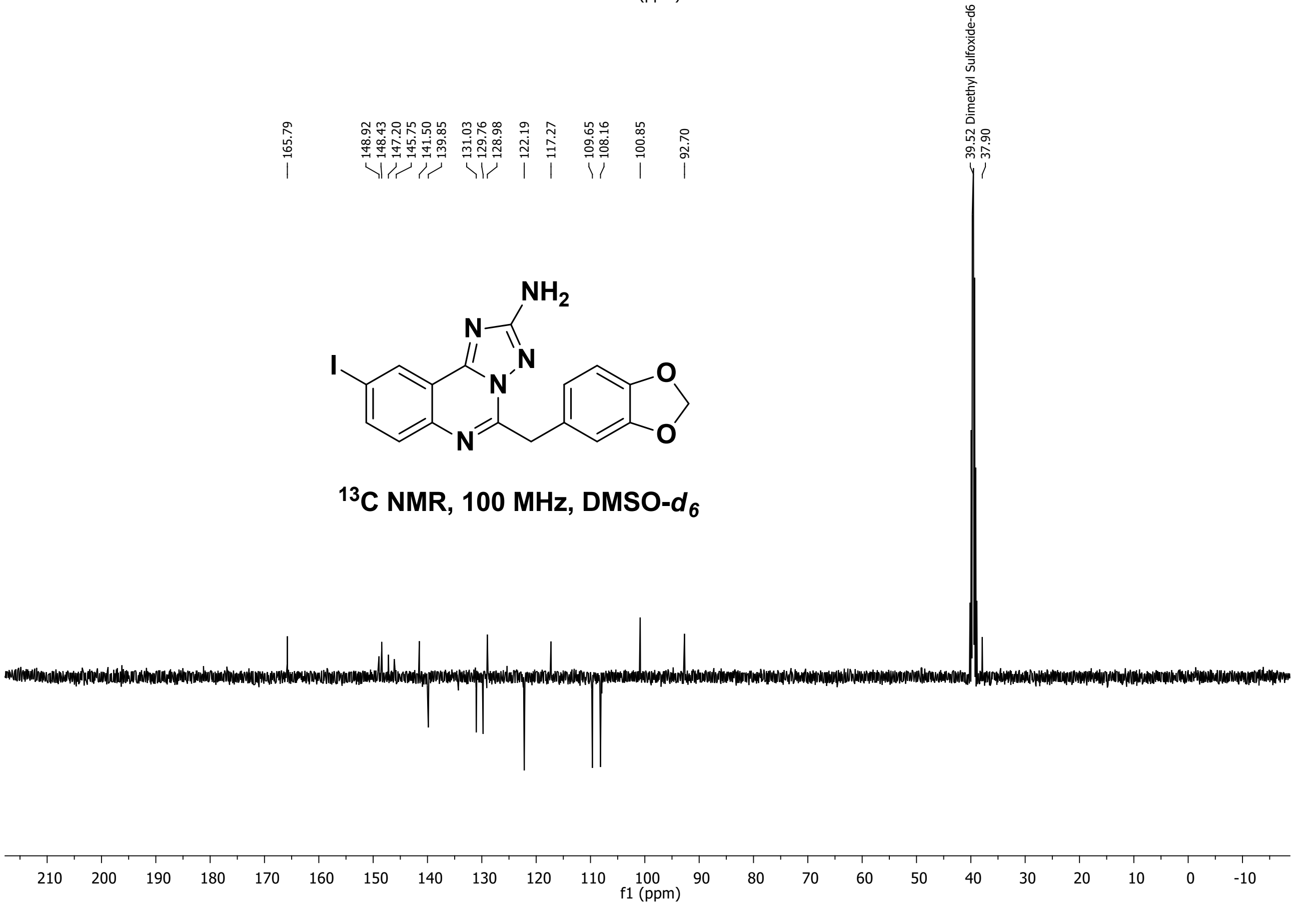

165.79
148.92
148.43
147.20
145.75
141.50
139.85
131.03
129.76
128.98
122.19
117.27
109.65
108.16
100.85
92.70
39.52 Dimethyl Sulfoxide-d6
37.90
NH2
N
N
N
I
O
O
N
13C NMR, 100 MHz, DMSO-d6
210 200 190 180 170 160 150 140 130 120 110 100 90 80 70 60 50 40 30 20 10 0 -10
f1 (ppm)

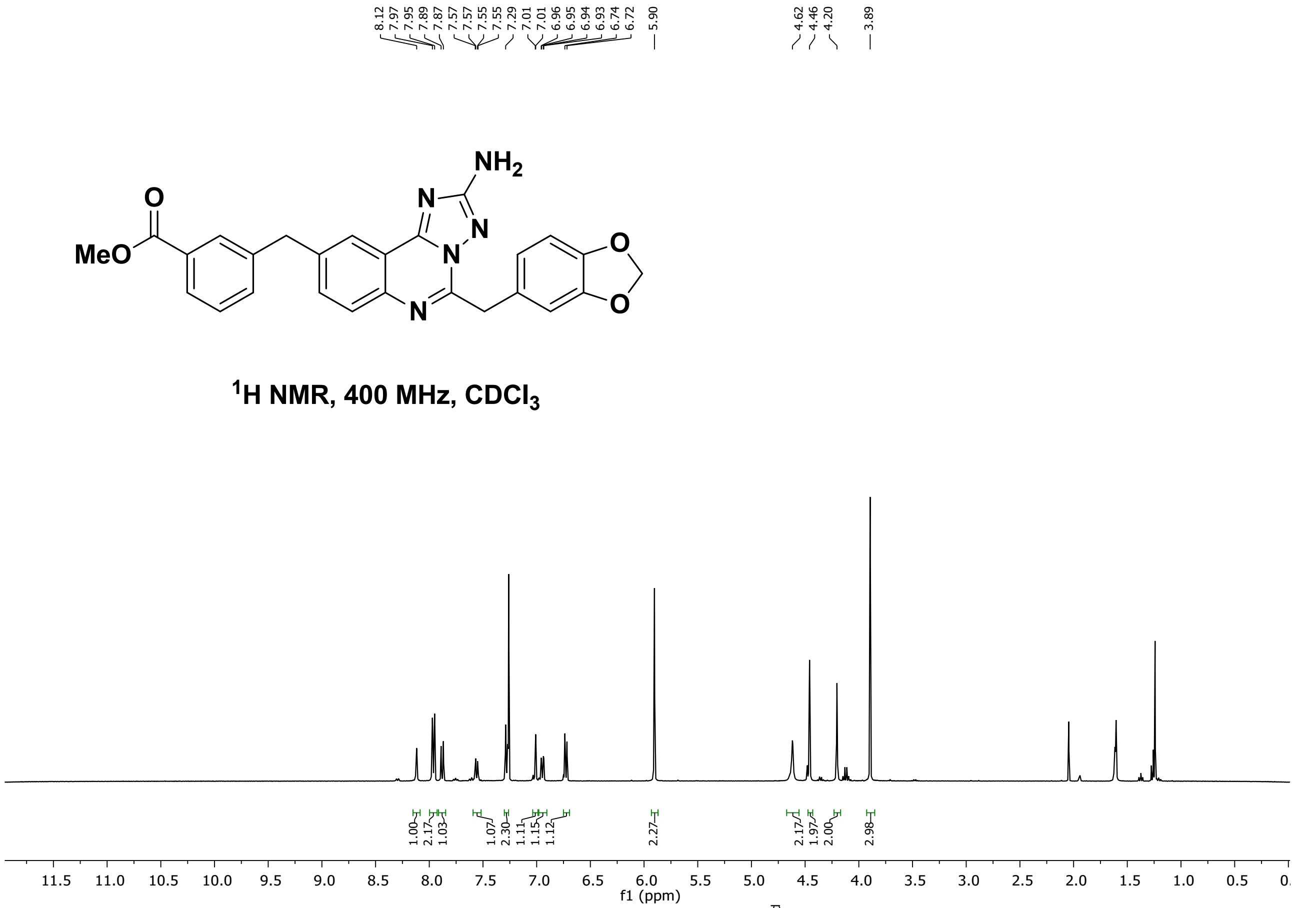
8.12
7.97
7.95
7.89
7.87
7.57
7.57
7.55
7.55
7.29
7.01
6.96
6.95
6.94
6.93
6.74
6.72
5.90
4.62
4.46
4.20
3.89
NH2
O
MeO
N
N
N
N
O
O
1H NMR, 400 MHz, CDCl3
1.00
2.17
1.03
1.07
2.30
1.11
1.15
1.12
2.27
2.17
1.97
2.00
2.98
f1 (ppm)

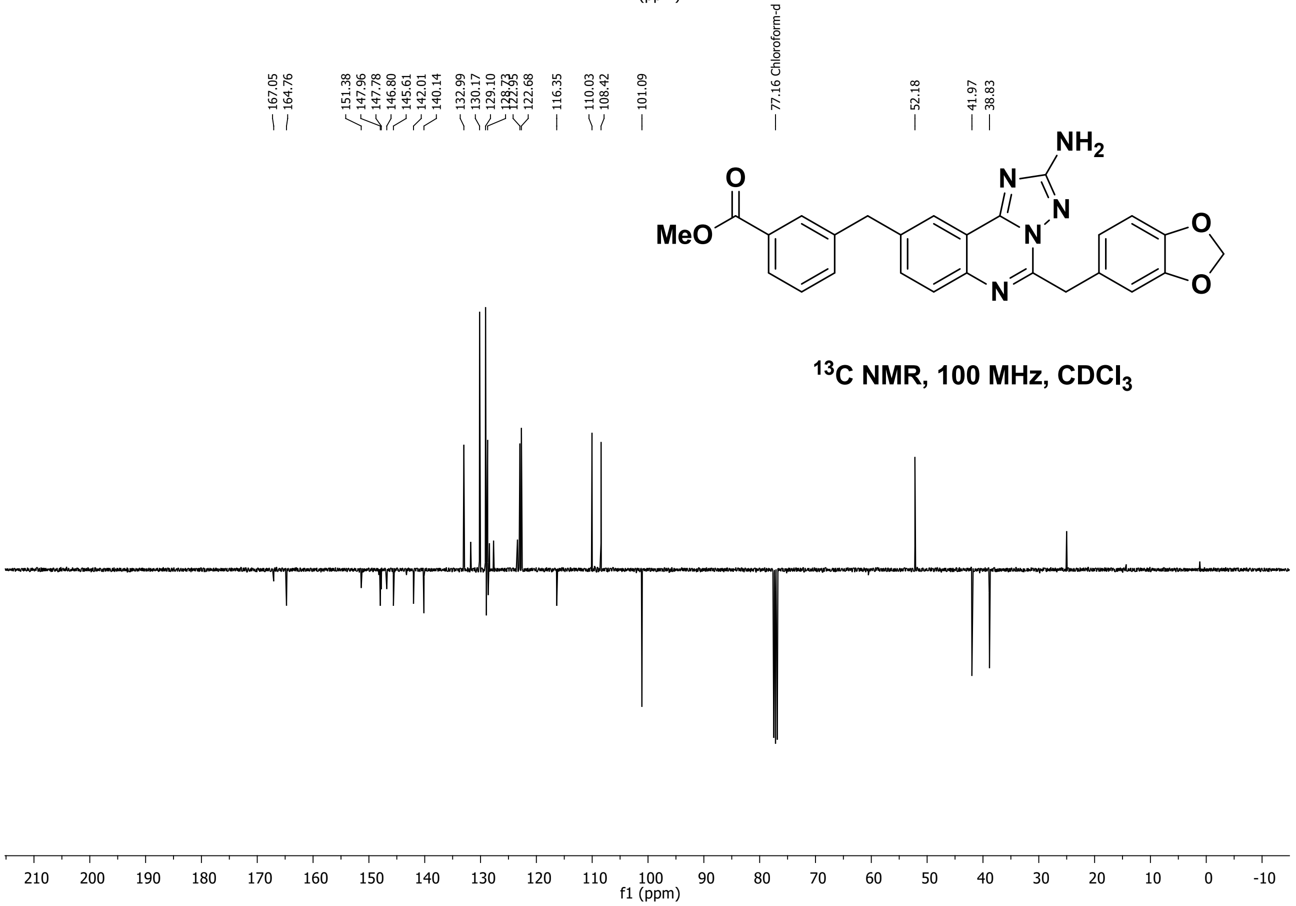
167.05
164.76
151.38
147.96
147.78
146.80
145.61
142.01
140.14
132.99
130.17
129.10
128.73
122.95
122.68
116.35
110.03
108.42
101.09
77.16 Chloroform-d
52.18
41.97
38.83
NH2
O
MeO
N
N
N
N
O
O
13C NMR, 100 MHz, CDCl3
f1 (ppm)

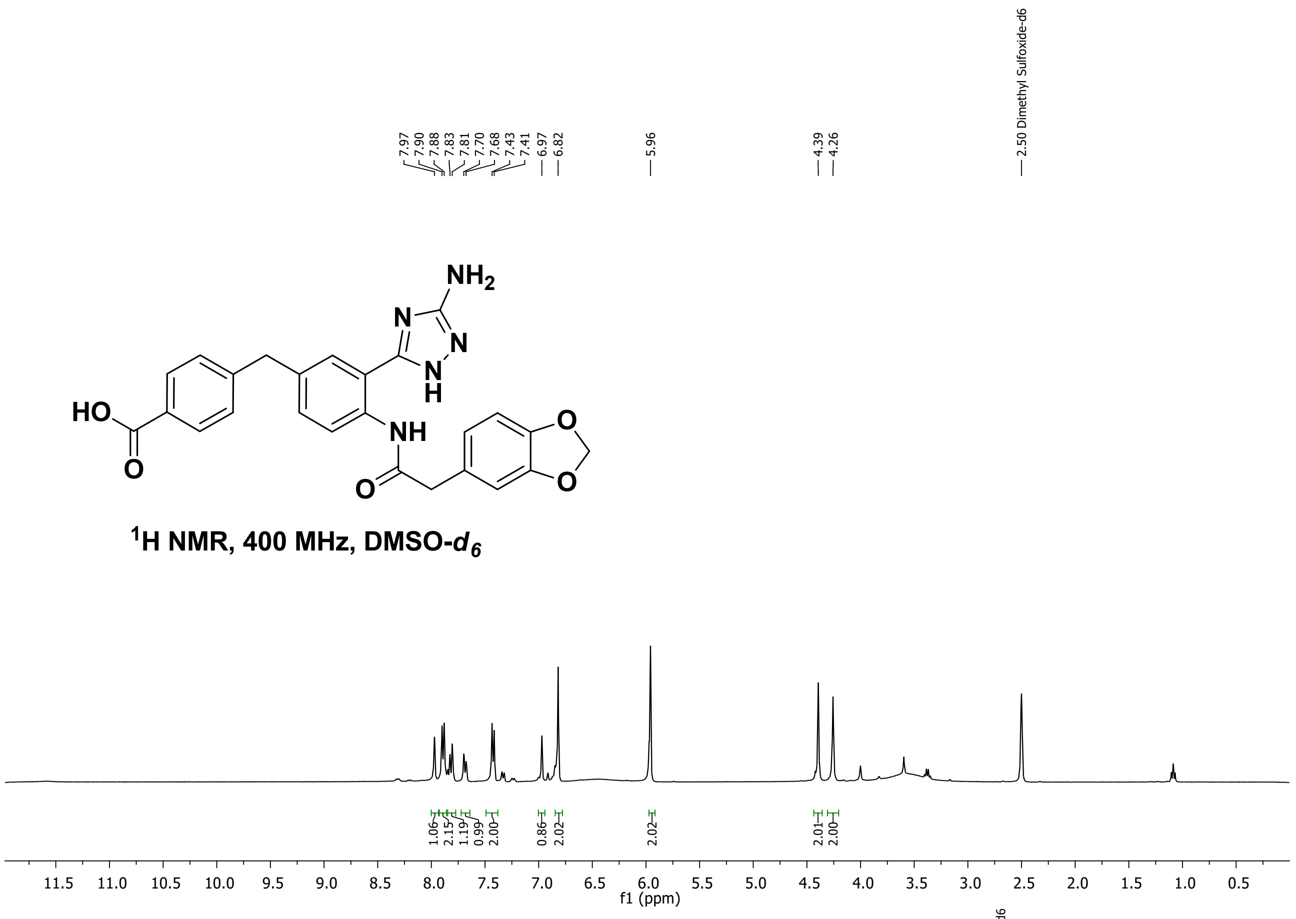
7.97
7.90
7.88
7.83
7.81
7.70
7.68
7.43
7.41
6.97
6.82
5.96
4.39
4.26
2.50 Dimethyl Sulfoxide-d6
NH2
N
N
N
H
HO
O
NH
O
O
O
1H NMR, 400 MHz, DMSO-d6
1.06
2.15
1.19
0.99
2.00
0.86
2.02
2.02
2.01
2.00
11.5
11.0
10.5
10.0
9.5
9.0
8.5
8.0
7.5
7.0
6.5
6.0
5.5
5.0
4.5
4.0
3.5
3.0
2.5
2.0
1.5
1.0
0.5
f1 (ppm)

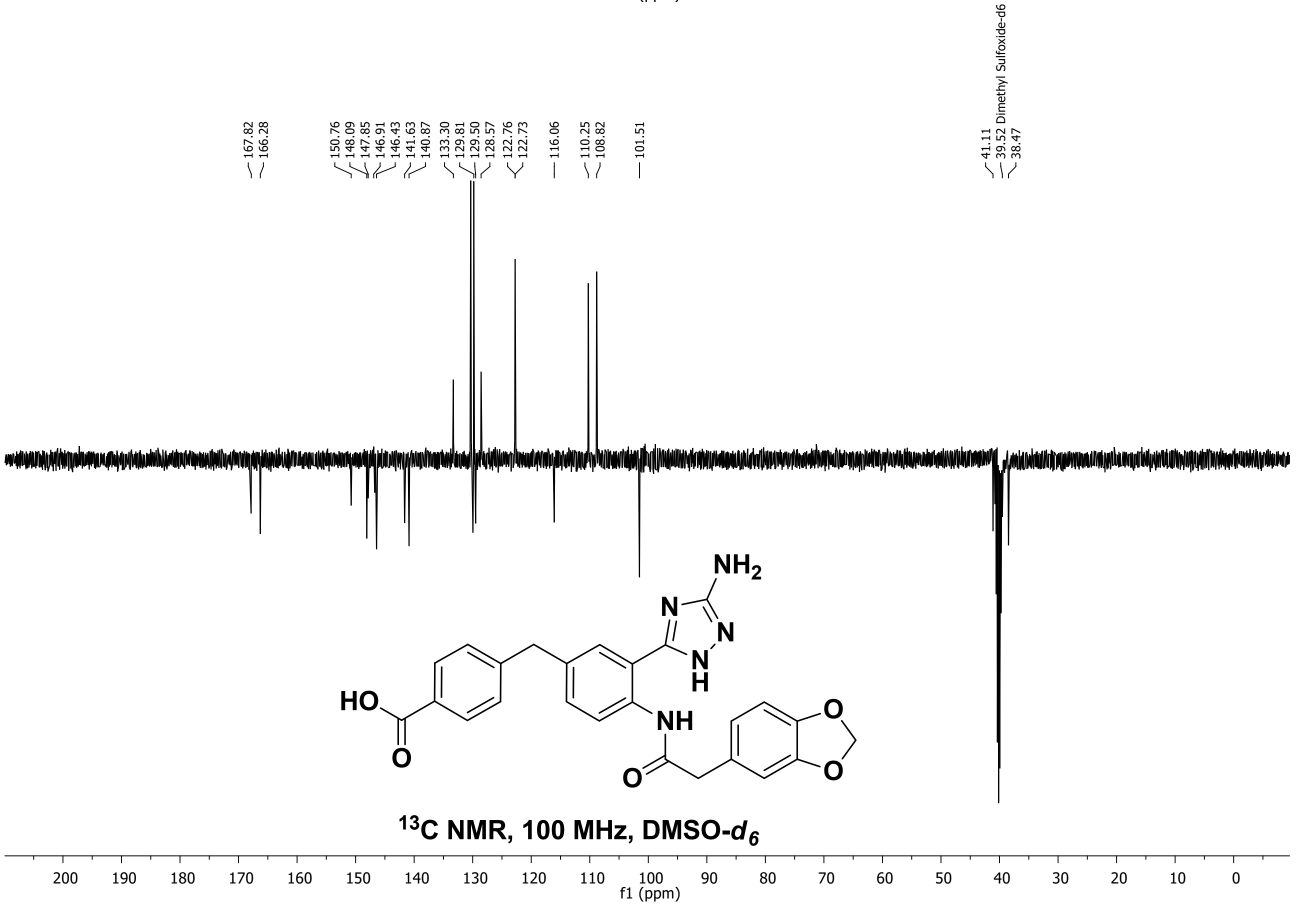
167.82
166.28
150.76
148.09
147.85
146.91
146.43
141.63
140.87
133.30
129.81
129.50
128.57
122.76
122.73
116.06
110.25
108.82
101.51
41.11
39.52 Dimethyl Sulfoxide-d6
38.47
NH2
N
N
N
H
HO
O
NH
O
O
O
13C NMR, 100 MHz, DMSO-d6
200
190
180
170
160
150
140
130
120
110
100
90
80
70
60
50
40
30
20
10
0
f1 (ppm)

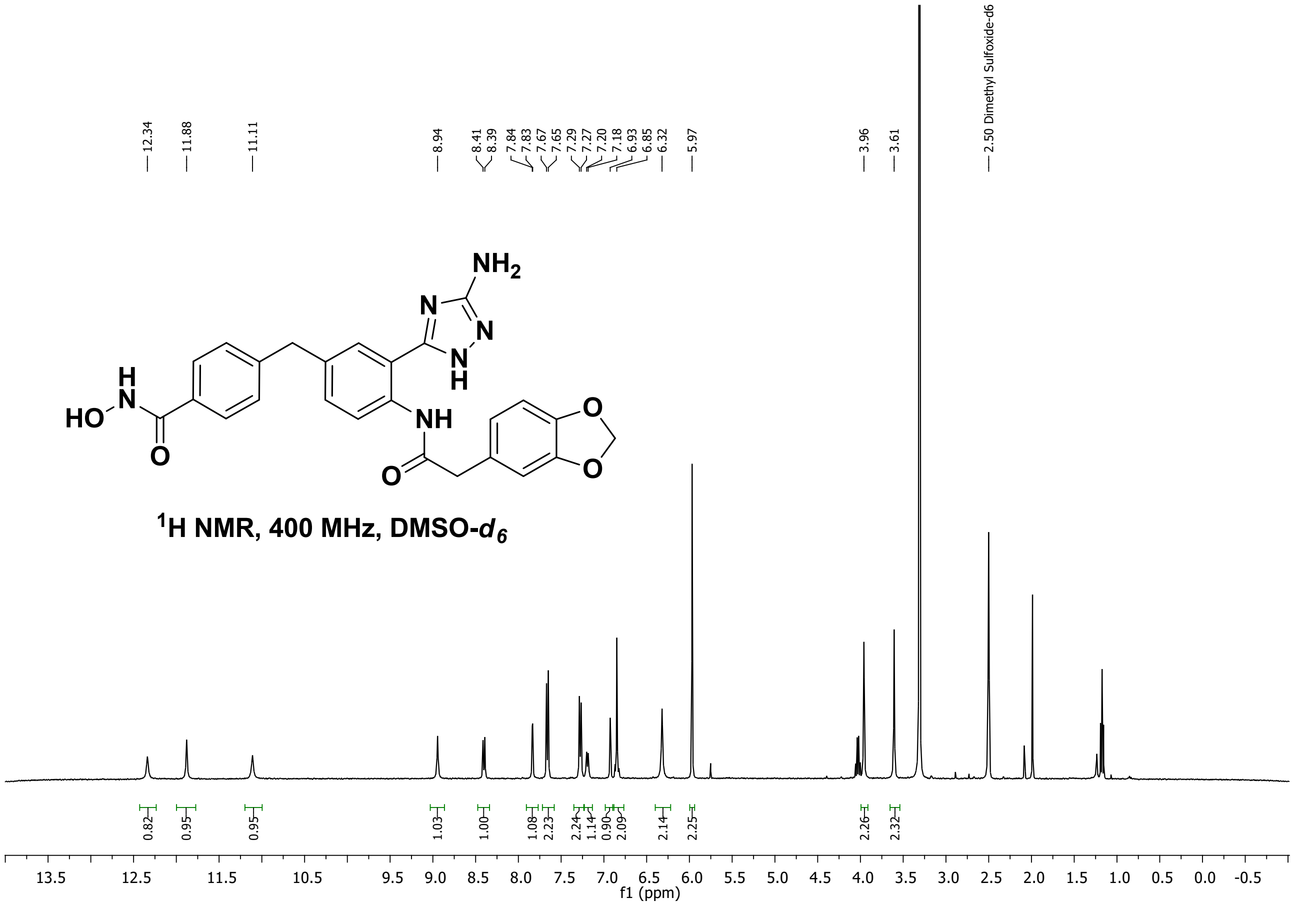
12.34
11.88
11.11
8.94
8.41
8.39
7.84
7.83
7.67
7.65
7.29
7.27
7.20
7.18
6.93
6.85
6.32
5.97
3.96
3.61
2.50 Dimethyl Sulfoxide-d6
NH2
N
N
N
H
H
N
HO
O
NH
O
O
O
1H NMR, 400 MHz, DMSO-d6
0.82
0.95
0.95
1.03
1.00
1.08
2.23
2.24
1.14
0.90
2.09
2.14
2.25
2.26
2.32
f1 (ppm)

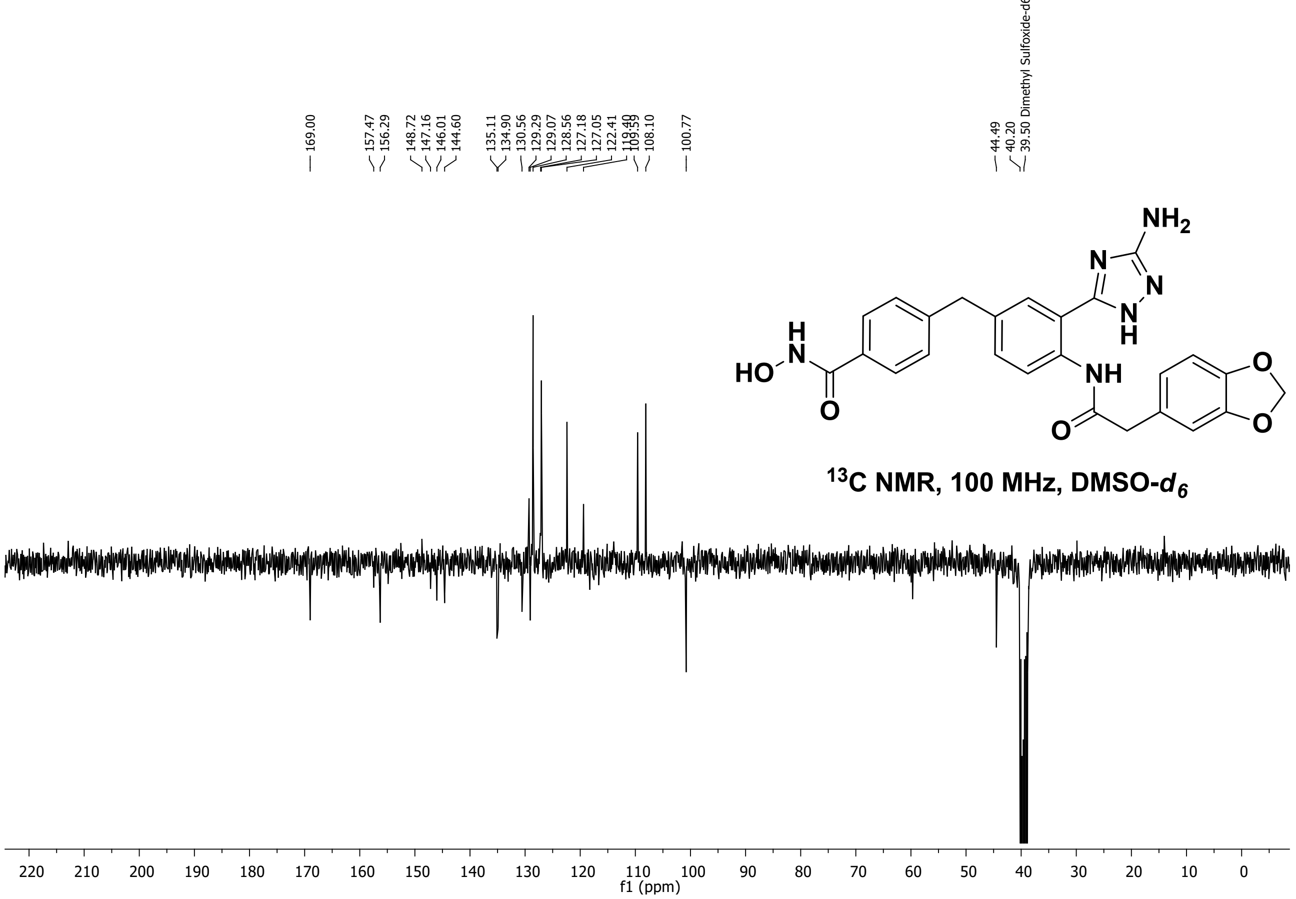
169.00
157.47
156.29
148.72
147.16
146.01
144.60
135.11
134.90
130.56
129.29
129.07
128.56
127.18
127.05
122.41
119.40
109.59
108.10
100.77
44.49
40.20
39.50 Dimethyl Sulfoxide-d6
NH2
N
N
N
H
H
N
HO
O
NH
O
O
O
13C NMR, 100 MHz, DMSO-d6
f1 (ppm)

HPLC-MS for 11a and 18

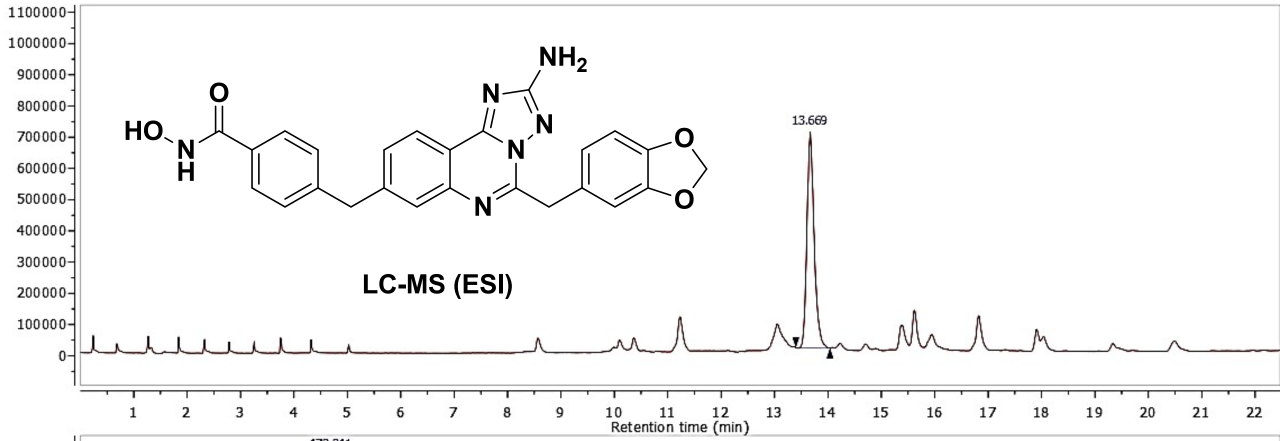

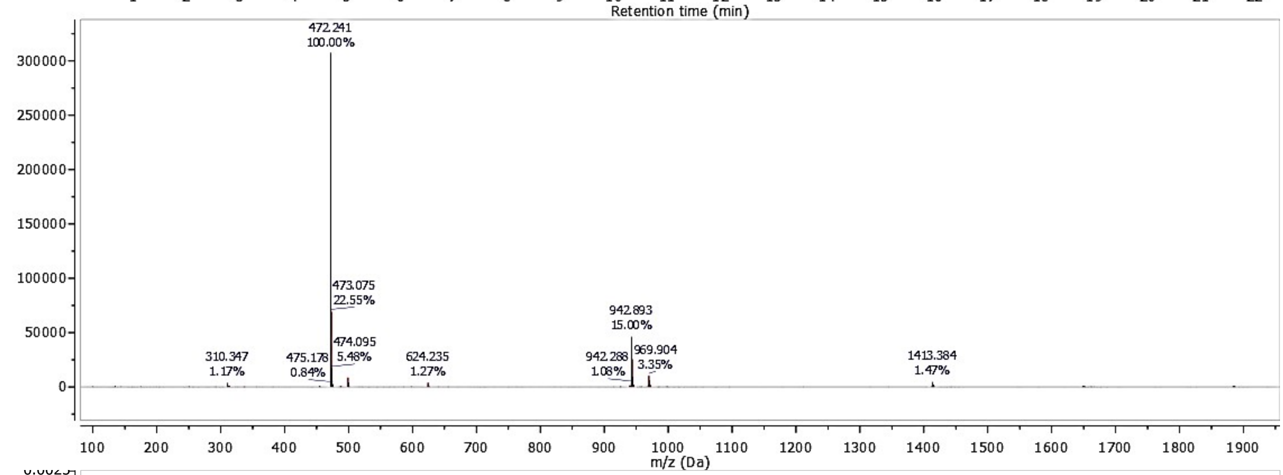

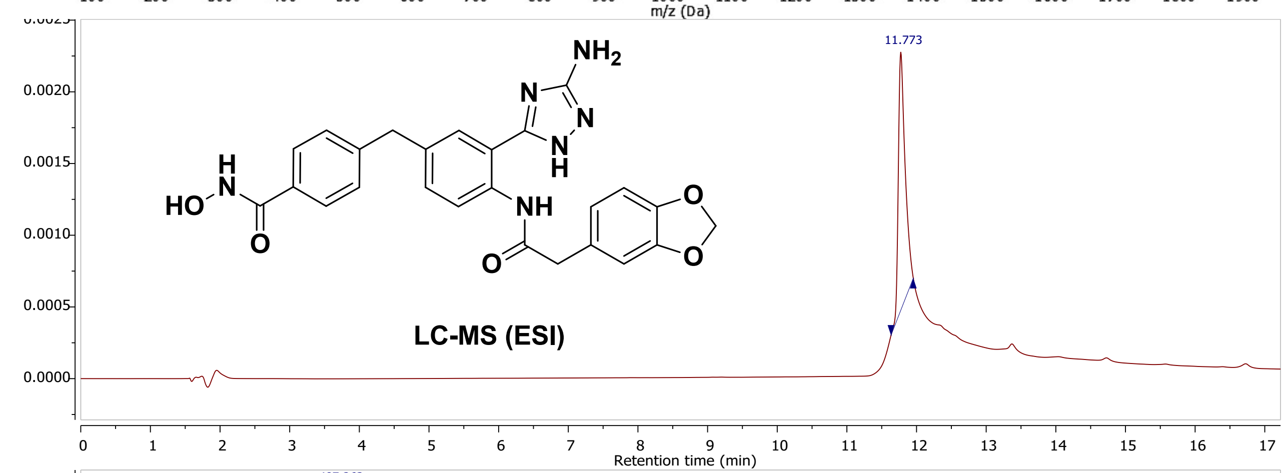

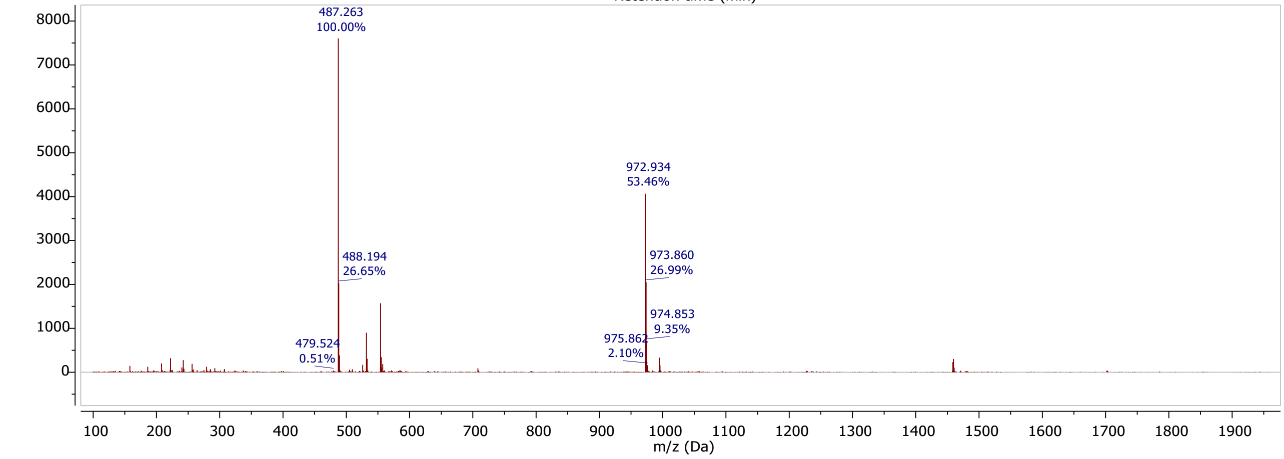

Supplement: RA-012-D2RA01753A-s001 [file RA-012-D2RA01753A-s001.pdf]
